# Contrastive Verbal Guidance: A Beneficial Context for Attention To Events and Their Memory?

**DOI:** 10.1111/cogs.70096

**Published:** 2025-08-14

**Authors:** Amit Singh, Katharina J. Rohlfing

**Affiliations:** ^1^ Psycholinguistics Paderborn University

**Keywords:** Event cognition, Contrastive guidance, Attention, Negation, Eye tracking

## Abstract

Research suggests that presenting an action via multimodal stimulation (verbal and visual) enhances its perception. To highlight this, in most studies, assertive instructions are generally presented before the occurrence of the visual subevent(s). However, verbal instructions need not always be assertive; they can also include negation to contrast the present event with a prior one, thereby facilitating processing—a phenomenon known as contextual facilitation. In our study, we investigated whether using negation to guide an action sequence facilitates action perception, particularly when two consecutive subactions contrast with each other. Stimuli from previous studies on action demonstration were used to create (non)contrastive actions, that is, a ball following noncontrastive and identical (Over–Over or Under–Under) versus contrastive and opposite paths (Over–Under or Under–Over) before terminating at a goal location. In Experiment 1, either an assertive or a negative instruction was provided as verbal guidance before onset of each path. Analyzing data from 35 participants, we found that, whereas assertive instructions facilitate overall action recall, negating the later path for contrastive actions is equally facilitative. Given that action goal is the most salient aspect in event memory due to goal‐path bias in attention, a second experiment was conducted to test the effect of multimodal synchrony on goal attention and action memory. Experiment 2 revealed that when instructions overlap with actions, they become more tailored—assertive instructions effectively guide noncontrastive actions, while assertive–negative instruction particularly guides contrastive actions. Both studies suggest that increased attention to the goal leads to coarser perception of midevents, with action‐instruction synchrony modulating goal bias in real‐time event apprehension to serve distinct purposes for action conceptualization. Whereas presenting instructions before subactions attenuates goal attention, overlapping instructions increase goal attention and reveal the selective roles of assertive and negative instructions in guiding contrastive and noncontrastive actions.

## 1 Introduction

**Fig. 1:**
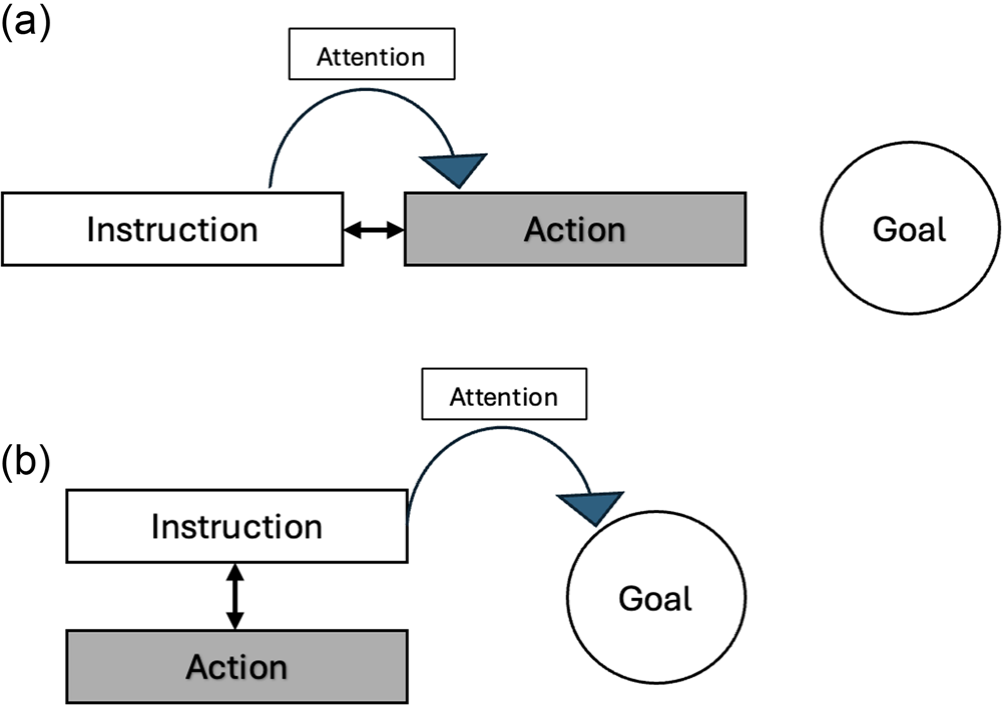
Two forms of multimodal synchrony: (a) sequential and (b) simultaneous. In the sequential presentation (Experiment 1), attention is directed toward the immediate action (path) as the instruction precedes and introduces the action. In the simultaneous presentation (Experiment 2), overlapping instruction and action are expected to shift attention directly to the action goal due to ever‐present goal bias in event perception.

Negation is ubiquitous in all the languages which enables us to talk about nonexistence, express rejection, denial, and impose prohibition (Choi, 1988; Horn, 2001). For example, an utterance “It will not rain today” let us express our belief that a particular event (namely: rain) will not occur. Given sufficient context, a listener might also be convinced to make an inference that it had been raining for the past few days but not today. In this way, negation allows speakers to convey something which counters listener's expectation (Choi, 1988; Drozd, 1995; Dale & Duran, 2011; Glenberg, Robertson, Jansen, & Johnson‐Glenberg, 1999; Lüdtke & Kaup, 2006; Orenes, Beltrán, & Santamaría, 2014; Stern, 1931; Wason, 1965). Thus, the use of negation seems to be highly context‐dependent (Albu, Tsaregorodtseva, & Kaup, 2021; Kaup & Dudschig, 2020), where one aims to contrast the present with the past event(s) (Glenberg et al., 1999; Horn, 2001). Although negation is among the most frequently used constructs in a language, it is often associated with difficulty in processing, false memory, and slower retrieval of concepts within its scope compared to affirmative counterparts (Kaup, Lüdtke, & Zwaan, 2006; Kaup, Zwaan, & Lüdtke, 2007; Mayo, Schul, & Rosenthal, 2014). In context of action performance, negation has been shown to cause inhibition at cognitive and neural level (Beltrán, Liu, & de Vega, 2021; de Vega et al., 2016). While studies have reported processing difficulty for sentential negations (Carpenter & Just, 1975; Clark & Chase, 1972), in the event cognition literature, negation remains an underexplored domain (Dudschig, Kaup, Liu, & Schwab, 2021), yet it could reveal promising consequences for action processing (e.g., Groß et al., 2023). In event processing research, it is known that the previous subevent acts as a context for the upcoming subevent, and in its entirety, event memory is more robust when the subevents are connected at some spatio‐temporal dimension (Zacks et al., 2001; Zacks & Swallow, 2007; Zacks, Swallow, Vettel, & McAvoy, 2006). The limited capacity of our attentional system highlights such importance of the coherence, that is, improved learning when modalities are unified across multiple levels—at verbal and visual (Bahrick, Lickliter, & Flom, 2004; Brand & Tapscott, 2007). It has also been found that participants often use negation to contrast and refer back to some previous subevent(s), such that something occurred previously but not this time (e.g., Tian, Ferguson, & Breheny, 2016). For such events, a verbal guidance which refers back to the previous subevent, while contrasting it with the present subevent, is more likely to facilitate a deeper connection at multimodal level. Linguistic negation is one such means which derives the meaning of an entity under its scope by referring to some alternate state of affair (Kaup et al., 2007; Talmy, 1975, 1985), thereby creating a rich pragmatic effect (Tian & Breheny, 2016). This unique feature highlights negation as a context‐generating constituent that bridges the present and the previous subevents (Zacks et al., 2006). For example, in a recent study, participants were shown a negative sentence either with or without a context contrasting an expected situation—for instance, *contrary to expectation, John did not iron his shirt* (Albu et al., 2021). In a probe recognition task, it was found that participants responded faster to a sentence when the expectation was contrasted with actual state of affair. Here, the later subevent “did not iron” might have been interpreted in light of the prior subevent “did iron,” which acted as a context, and thus eased its processing. It has also been shown that participants are more likely to negate a stimuli depicting an absence of a feature when surrounding stimuli already possess that particular feature (Nordmeyer & Frank, 2023; Wason, 1965). This suggests a situated use of negation, where an event in hand can be defined by the absence of a prior event or one occurring in close vicinity, especially when they are temporally or spatially bound. Based on these findings, it has been suggested that context can mitigate processing cost associated with negations (Lüdtke & Kaup, 2006). Nevertheless, the effect of contextual facilitation on negation has been studied mostly for linguistic tasks and has rarely been examined in the context of actions, which inherently incorporate temporality, that is, events that unfold continuously over time (Huff, Papenmeier, & Zacks, 2012; Zacks et al., 2001). The majority of studies in this area focus on how access of a concept is eased, when the surrounding context is static, or provided before a sentence. When both sentence processing and action performance are brought together, a close interplay between linguistic processes and event conceptualization is observed (Altmann & Kamide, 2004). Crucial to this interplay, one perspective argues that language influences even the very early event perception, guiding attention through its innate semantic structures, which becomes a part of procedural knowledge through language use (Levelt, 1993). Conversely, another view suggests that early attentional processes are guided by domain‐general mechanisms, wherein event conceptualization at the perceptual stage remains unaffected by the syntacto‐semantic structure of language and is only influenced at later stages (Griffin & Bock, 2000). Despite this temporal distinction, both perspectives agree that language directs attention to event structures acting as a spotlight (Wolff & Holmes, 2011). To capture these concurrent processes, online methods like eye tracking have been employed. For instance, studies particularly in motion event show that eye movements are consistently drawn more toward the event goal (path) than other event components, such as manner, regardless of whether linguistic or nonlinguistic processes are involved (Papafragou, 2010; Zacks & Swallow, 2007). From a developmental perspective, several lines of evidence indicate that infants can register predictability structure within human activity in relation to actors' goals, as opposed to just specific surface motion patterns actors adopt to pursue those goals (Kosie & Baldwin, 2021; Olofson & Baldwin, 2011; Woodward, 1998). They can also organize their processing of goal‐directed activity around that predictability structure by, for example, directing gaze to an actor's predicted goal object in advance to the actor's actual contact with that object (Ambrosini et al., 2013; Kanakogi & Itakura, 2011; Monroy, Gerson, & Hunnius, 2017). A common explanation for such behavior is that observing an action inherently engages the observer's own motor system, allowing them to simulate the action and anticipate the agent's goal (Gredebäck & Falck‐Ytter, 2015; Rizzolatti & Craighero, 2004). From a social interactional perspective, prevailing debates on how actions are learned have focused on whether, and to what extent, sensorimotor processes involved in goal anticipation are influenced by top‐down linguistic and semantic structures (Griffin & Bock, 2000; Papafragou, Hulbert, & Trueswell, 2008; Wolff & Holmes, 2011). This issue was approached by investigating processes related to language‐mediated sensorimotor behavior, specifically testing whether language can operate on sensorimotor processes to influence event and action apprehension (Holmes & Wolff, 2010; Wolff & Holmes, 2011). The mechanisms through which language (verbal behavior) may meddle with actions can also be sensitive to how the modalities are aligned (Brand & Tapscott, 2007; Bahrick et al., 2004). In situations where language is an ostensive cue and acts as a spotlight, directing attention to the immediate subaction may mitigate the goal bias arising from inherent sensorimotor processes. A sensitivity to ostensive cues alerts learners that there is an opportunity to learn, and accordingly, important aspects are communicated (Csibra & Gergely, 2009). This is a well‐documented phenomenon; for example, Nomikou and Rohlfing (2011) found that mothers used verbal instructions prior to a particular subaction to introduce and highlight it, thereby making those subactions salient in relation to the action goal (see also, Sciutti, Lohan, Gredebäck, Koch, & Rohlfing, 2016). In an alternate view, early learning has also been shown to be shaped by co‐occurrence of visual and verbal information, such as, through modality overlapping (Bahrick & Lickliter, 2000, 2002). When modalities overlap, language creates sensorimotor redundancy that facilitates the detection of amodal properties of the stimulus (Bahrick et al., 2004). The two ways of multimodal correspondence between language and action may allude to different forms of instruction‐action synchrony. Whereas presenting instructions prior to a subaction may subserve the learning of fine‐grained aspects of an action by spotlighting elements that might otherwise go unnoticed, overlapping modalities may instead lay the foundation for action structuring through goal‐directed component. Hence, considering the possible ways in which language may structure action perception, we conducted two eye tracking experiments to test two forms of instruction‐action synchrony: presenting instructions either before or concurrently with actions (Fig. 1). The primary objective of this study was to ground negation in an action performance task to determine whether negation, as a form of contrast, provides plausible context for action understanding. Subsequently, we aim to examine whether, and to what extent, action contexts are sensitive to synchrony of the modalities involved, in light of the ever‐present goal bias in event memory. These findings can have implications for effective verbal guidance in many instructional settings, such as learning or working together.

### 1.1 Grounding negation in action performance

Although previous studies have provided limited focus on the interplay between negation and action, they are nonetheless informative about the underlying cognitive mechanisms that our investigation can capitalize upon. Recent studies have found that negation leads to action inhibition (Beltrán et al., 2021; de Vega et al., 2016), for which the effect could go back to alternate state of affair being activated before the actual state of affair (Kaup et al., 2006; Kaup et al., 2007). This proposition is made in early studies showing that a two‐step mental simulation takes place as someone encounters a negated sentence. For example, in a study by Kaup et al. (2007), participants were shown a sentence, such as “There was no eagle in the sky,” and thereafter, a picture matching a negated state of affair (an eagle with stretched wings) or an actual state of affair (an eagle with folded wings). It was found that after a short Stimulus Onset Asychrony (SOA) of 500 ms, participants responded faster to a picture matching the negated state of affair than to a picture matching an alternate state of affair. Crucially, this pattern was reversed when a sufficient time for response was provided, which presumably allowed participants to simulate the situation after reading the sentence. The results suggested that participants relied predominantly on mental simulation while processing the negative sentences. Based on findings like this, the conceptual access under negation has been shown to be governed by a two‐step cognitive process, where at the first step, the activation is guided by the propositional structure, and at the later, by the situational model (Kaup et al., 2006; Kaup et al., 2007). This simulation has been shown to be constitutive of the domain general cognitive processes (Mayo et al., 2014), which directly connects to actions and embodiment accounts (Barsalou, 1999; Glenberg & Gallese, 2012). For the sake of completeness, it should be noted that other studies, however, proposed rather a single step account (e.g., Lüdtke & Kaup, 2006; Tian et al., 2016). It is suggested that negation also relates to sensorimotor system, which involves general cognitive mechanism, such as response inhibition (de Vega et al., 2016). More specifically, in an electroencephalography (EEG) study by de Vega et al. (2016), participants were asked to read an assertive or a negative sentence while doing a NoGo task; it was found that theta oscillation—a neural marker of response inhibition—was smaller in the negative than the assertive condition. Notably, the studies in favor of two‐step cognitive process usually focus on picture recognition, inhibitory control, or sentential negation, where the accessibility of a critical concept is measured through recording of the reaction time and accuracy. Accordingly, a shorter latency then corresponds to easier access to the concept under consideration, and the effect of context, especially in an action performance task, is yet to be explored. If negation processing links to a domain‐general processing mechanism, which relies on simulation and the contextual factors, then measuring these effects directly for action performance, such as in an action recall task, provides a promising area of investigation.

### 1.2 Action and attention to the goal

We argue that negation, although mostly studied as a linguistic means, also connects deeply to actions (Bartoli et al., 2013; Tomasino, Weiss, & Fink, 2010; Tettamanti et al., 2008). For example, negation is found to be crucial in action learning during developmental stage (Bloom, 1993; Horn, 2001). It is a fundamental feature to elicit response inhibition verbally in children, for example, saying “No!” for an action not only inhibits the current response, but also acts as a scaffolding means, where correct response thereafter can implicitly or explicitly be conveyed (Bloom, 1970; Drozd, 1995). Diverting learners' attention from a specific entity using negations and guiding it to the relevant aspects of the task is directly bound to the task goal. This method is frequently used by the caregivers toward a developing child in an interaction, be it for language acquisition (Bloom, 1970; Choi, 1988; Drozd, 1995; Hummer, Wimmer, & Antes, 1993; Tagliani, Vender, & Melloni, 2022) or action learning (Bloom, 1993; Woodward & Sommerville, 2000).

But what makes actions a special case for negations? It is now well known that human behaviors, including actions, are goal‐directed (Zacks et al., 2001). This goal is maintained at a higher level of availability and is foregrounded from other constituents in an action (Ikegami, 1979; Talmy, 1985; Zacks et al., 2001). For example, infants have been shown to structure their actions in relation to goals (Kosie & Baldwin, 2021), and studies with adults report a better memory for goals than for other components—such as source—in a motion event (Ikegami, 1979; Papafragou, 2010; Talmy, 1975). In one study, participants were shown an animate object moving from a source to a goal location, after which a change in goal or source was probed. It was found that participants were more likely to report a change in the goal than the source location, suggesting that source is not encoded to the same extent as goal in a motion event (Papafragou, 2010). Additionally, in a motion event, there are two fundamental components identified: path and manner (Slobin, 1996; Talmy, 1975). In this context, the path characterizes “Where” component in a motion, while the manner specifies “How” the action should be performed. The path has also been associated with goal of a motion event, toward which the path terminates at the end (Talmy, 1985, 2000). Implementing this idea within an interactive framework, a related study using human–robot dialogue found that when either path or manner information was missing, path information was sought more quickly than manner information to complete the task at hand, suggesting that bias effect can be also elicited by the path component in actions (Singh & Rohlfing, 2024). The prioritization of goal in event cognition, while utmost beneficial for task in hand, may potentially undermine the attention on fine‐grained intermediary subevent(s) within a continuous action. For instance, a multitude of studies (also in infancy research) have consistently demonstrated a coarse perception of manner than path (Bunger, Skordos, Trueswell, & Papafragou, 2021; Hespos, Grossman, and Saylor, 2010; Papafragou et al., 2008). And the manner in which an action is performed is not captured to the same level as the path or trajectory (Hespos, Saylor, and Grossman, 2009; Skordos et al., 2020). Thus, to optimally learn from action demonstrations, it is crucial that the path and manner receive a comparable degree of attention. In such a situation, highlighting the manner in an action, and simultaneously reducing the attention from goal using a verbal means (or perhaps negation), might be beneficial for a better action conceptualization. In this respect, from a very recent study on attention, we can draw potential for negation in facilitating a more even distribution of weights across stimuli (Banh, Tünnermann, Rohlfing, & Scharlau, 2024). However, in this study, the context of negation was not systematically varied and was applied solely to static stimuli within the spatial domain, without visual contrasts. Unlike static events, a continuous unfolding action that elicits a temporally bound contrast between two subevents may offer a more suitable context for examining the effects of negation. Whereas negation has the potential to meddle with sensorimotor processes through its unique property of inhibitory processes (de Vega et al., 2016; Kaup & Dudschig, 2020), language itself has a broader propensity to affect early sensorimotor processes by spotlighting specific aspects—those that might otherwise go unattended due to a developmentally learned routines of goal prominence (Sciutti et al., 2016; Wolff & Holmes, 2011). Most of this learning takes place during early childhood through multimodal synchrony (Bahrick & Lickliter, 2000; Bahrick et al., 2004). Essentially, the tight temporal co‐occurrence of verbal and action routines plays a key role in decoding the “semantics” of the action, guiding it through goal interpretation (Bahrick et al., 2004). Verbal instructions that are not strictly overlapping with actions but are instead introduced beforehand may meddle with this learned behavior, potentially reorienting or sustain attention to elements that would typically go unnoticed in continuous actions. Hence, synchronizing instructions in such a way that language acts as a spotlight—not for the most salient aspect, but on coarser, intermediate aspects of the action—may hold potential for inducing subtle but meaningful effects in instructional settings (Fig. 1).

## 2 Current study

The present study primarily aimed to assess the effect of context induced by negation on the perception of contrastive and noncontrastive actions. For this, we used simple action models in which a contrast is achieved on two action paths following each other. The participants had to recall these constellations. To elicit a contrast, the task would need to satisfy following requirements: (A) The contrast must occur at least at one of the components of action, namely, the path or manner, and (B) the present action component should be opposite to the prior action component on some spatio‐temporal dimension. Given these requirements, the stimuli were adopted from a previous study on action learning in infants (Hespos et al., 2009; Hespos et al., 2010), where the path direction was manipulated. We selected these stimuli because they consisted of a spatial framework at the most salient dimension of path manipulation in vertical direction (e.g., Over and Under), which is salient than horizontal (Front–Back or Left–Right) due to ever present effect of gravity (Clark, Carpenter, & Just, 1973). In addition, we combined verbal guidance in assertive and negative forms to guide these paths. Overall, we expected to observe an inverse relationship between attention to the goal and action recall since this aligned with our prediction that there exists a general trade‐off between attention to the goal and memory of the midevent constituents (e.g., manner) in the action demonstration (see Section 1.2). Our primary hypothesis relates to testing an action context that permits the use of negation. A plausible context is defined when the negation denies the expected subevent or indicates the absence of a previous subevent, such that something previously happened but not this time. We reasoned that such contexts would facilitate a rich connection between the prior and the current subevents at two levels (verbal and visual). For example, in a contrastive action, when the later path is opposite of the previous path (Over–Under), using negative guidance for the later path (e.g., NOT Over) not only de‐biases from the expectation but also refers to the previous path (Over). This “back referencing” allows a connection at the verbal level as compared to the simple assertive guidance (Now Over–Now Under), which only facilitates connection at the visual level and misses the opportunity to utilize the previous context. Thus, we predicted a better memory and attenuation of attention from the goal for the contrastive action in the presence of an assertive–negative instruction. We also predicted an overall higher recall for an action when it is guided through assertions as compared to when no guidance is provided (a baseline condition), because a multimodal presentation of an action has been shown to facilitate its perception (Bahrick & Lickliter, 2000, 2002; Brand & Tapscott, 2007). This is especially the case when an action comprises of repetitive paths or subactions without contrasting each other (noncontrastive). Hence, for noncontrastive actions, we expected higher recall and goal attenuation under assertive guidance, as negation would be implausible in the absence of visual contrast—making simple assertive guidance the most facilitative. Accordingly, we also predicted lower action recall and higher attention to the goal when a negation is used to guide a noncontrastive action, for example, a guidance like [Now Over–NOT Under] used for two upward motions. Overall, we tested all possible combinations of assertive and negative guidances on contrastive and noncontrastive actions, each comprising two paths following each other.

Previous studies have also emphasized the sensitivity of verbal–action temporal synchrony on learning and attention (Bahrick & Lickliter, 2000, 2002; Bahrick et al., 2004; Brand & Tapscott, 2007). Building on this, we tested our hypothesis across two experiments: instructions presented before the action (Experiment 1) and instructions presented simultaneously with the action (Experiment 2). The two forms of temporal synchrony were found to influence attention to the modalities in distinct ways. A sequential verbal–visual presentation has been frequently employed when the goal is to introduce and spotlight a particular subaction, as observed in infant–mother interactions (Nomikou & Rohlfing, 2011). Furthermore, it is suggested that unimodal stimulation promotes prolonged and sustained attention to the modality in question, making its properties more salient, as only one of the two modalities is present at any given time (Bahrick et al., 2004). Thus, for sequential presentation, we predicted that when instructions precede actions, sustained attention on the immediate event would delay the attention on the goal (as in Fig. 1a). In contrast, when instruction and action overlap (Fig. 1b), the modalities are perceived as unitary due to a well‐established phenomenon of intersensory redundancy for which our cognition is developmentally shaped (Bahrick & Lickliter, 2000, 2002; Bahrick et al., 2004). In such cases, the default behavior of our perceptual system is to prioritize the action goal—unless, ostensive attention is directed toward the upcoming action, as in sequential presentation (Fig. 1a). Therefore, in this condition, we predicted that the attention would gravitate directly toward the action goal, which serves as the default locus of attention (Lakusta & Landau, 2012; Zacks et al., 2001).

## 3 Experiment 1: Instruction before the action

### 3.1 Method

#### 3.1.1 Participants

The sample size for the study was determined by the previous eye tracking studies on the event understanding (Bunger, Skordos, Trueswell, & Papafragou, 2016; Papafragou et al., 2008). Additionally, a post‐hoc power analysis was conducted on pilot study data.^1^ Our goal was to achieve 0.95 power (95%) to detect a medium effect size of 0.25, with a standard alpha error probability of 0.05, which suggested a sample size of 30. Thirty‐five university students (mean age = 23.90, *SD* = 2.97) who were native or fluent German speakers took part in the experiment. An informed consent was obtained prior to the data collection in accordance to the university ethical board. All the participants were recruited through classroom advertisement and were compensated either a course credit or remuneration for their participation. Data from three participants (*N* = 3) were excluded from the analysis, due to the track‐loss or failing to properly follow the experiment instruction. Thus, the final sample size for this experiment was 32.

#### 3.1.2 Stimuli

**Fig. 2:**
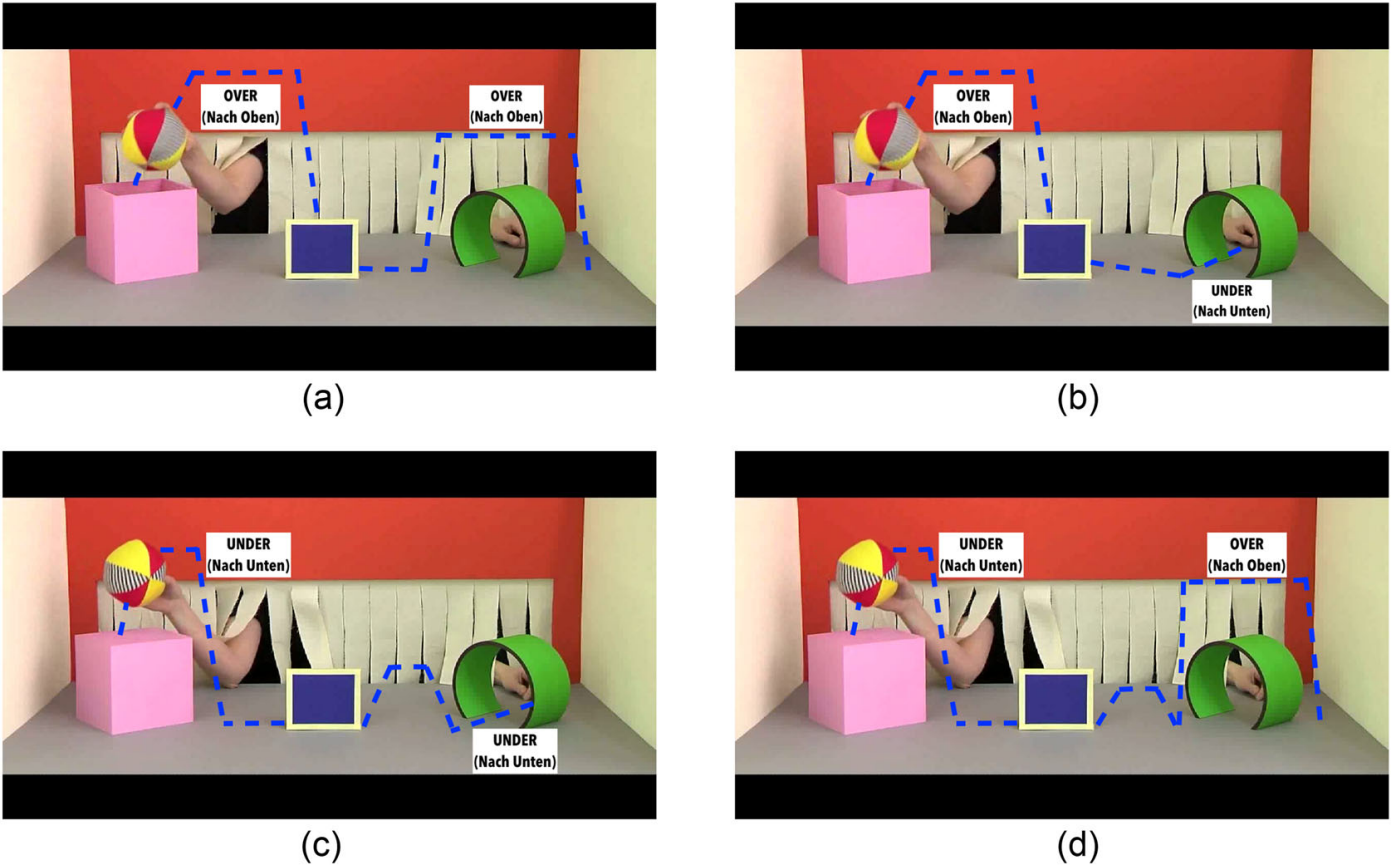
Stimuli depicting the action conditions. Blue line shows the path of the ball with instruction in assertive condition.

The study replicated four videos from Hespos et al. (2009); Hespos et al. (2010), depicting a ball moved from a source toward the goal. During this entire video, the ball showed two prominent changes in the path direction at the source and at the goal locations. These changes were characterized by an upward or a downward motion. A video in which the direction of the later path (at goal) contrasted the previous path (at source), we treated it as a contrastive action, for example, an upward path followed by a downward path or vice versa (Fig. 2b,d). Conversely, a video in which the direction of the later path (at goal) was a repetition of the previous path (at source), we treated it as noncontrastive action, for example, two upward or two downward paths in a sequence (Fig. 2a,c). Two types of contrastive and noncontrastive actions were created to represent the two levels of complexity. This was done to preserve some variability in the experimental items. Crucially, across these items, the nature of the action—contrastive versus noncontrastive—remained consistent, specifically involving changes in the path direction at the source and goal locations. A verbal instruction was used to guide the directions. These instructions were recorded by a female speaker in an intonation which is used while demonstrating an action. The guidance was provided in an assertive or a negative form, for instance, a guidance for the upward path direction in assertive condition was “Nach Oben!” [“Toward Over!” in English] or in negative condition “Nicht Unten!” [“Not Under!” in English]. Similarly, a downward path direction in assertive condition was “Nach Unten!” [“Toward Under!” in English] or in negative “Nicht Oben!”[“Not Over!” in English]. Crucially, the “Nach” and the “Nicht” correspond to a path preposition and negation particle, respectively. In a motion event, the path preposition like *toward (Nach)* refers to an execution of an action in the direction of a reference object (Talmy, 2000). Hence, we used this preposition to highlight the direction of the path in assertive condition and to make the conditions comparable in number of words. As a result, the instruction conditions for two consecutive segments in an action were: assertive–assertive, negative–negative, assertive–negative, and negative–assertive. A video without a verbal instruction was used as the baseline. A total of 20 trials were created by combining four videos and five instruction conditions including the baseline where the instruction was absent.

#### 3.1.3 Procedure

The experiment was designed on the 1920 X 1080 pixels monitor equipped with Tobii‐Pro 120 Hz eye tracker. The size of the video was 1080 x 720 pixels. Participants were seated on a swivel chair approximately 60 cm from the screen. The objects used in the video were placed behind the participants on a small stage for the recall of the actions. A video camera was installed behind the stage to record the action performance. The participants were introduced to the experiment and the setup. They were told that they will watch 20 videos one by one in a sequence. In these videos, a ball will be moved by a person on a similar stage which is placed behind. Each video will have a verbal instruction. The task is to carefully watch the video, remember the actions shown, and then perform it on the stage. The experiment started with a fixation followed by the first video then a probe text at the end. The probe asked the participants to turn back and perform the action shown in the video. Participants then turned back while seated, performed the action on the stage, and then returned back to the initial position to proceed with the next trial. During this entire trial, participants did not require to stand up from the chair. The videos were randomized and self‐paced where participants were given unlimited time to perform the task. The next trial was initiated by pressing the space bar on the keyboard. The position of chair was marked on the ground to maintain a fixed distance between the eye tracker and the participant. Prior to the main experiment, participants were given 10 practice trials to familiarize themselves with the experimental setup. In the practice session, the objects and its path direction were different from the main experiment to avoid any carry‐over effect. After the practice session, the main experiment began. The entire experiment lasted for approximately 30 min in which the eye gaze and video data were recorded.

#### 3.1.4 Analysis

For the recall coding, the videos of action demonstration and performance were exported from the eye tracker and video recorder, respectively. For the fixation metrics, the data was extracted from the Tobii‐Pro Lab software after preprocessing. All the analyses were done in R version 4.4.0 (R Core Team, 2022) and R studio v12.1.402 (Posit team, 2024).

##### 3.1.4.1 Preprocessing of eye movement data

A polygon Area of Interest (AOI) was created for the goal object (here: Green Ring). This was based on the previous studies suggesting that the goal receives maximum attention than any other entity within an action (Bunger, Trueswell, & Papafragou, 2012; Bunger et al., 2016, 2021; Papafragou et al., 2008). To avoid any coincidence of moving ball with the goal, the goal‐AOI was only active from the beginning of the trial until the ball reached the goal. This was consistent with the previous method used in event cognition to investigate the attention to the goal (Papafragou et al., 2008). Fixations were classified using Tobii‐Pro‐IV inbuilt filter. For a time‐dependent analysis, each trial was divided into 200 ms time bins following the previous research showing that saccades approximately take 200 ms to launch (Saslow, 1967). To determine the fixation proportion in each time bin, the number of fixations falling in an AOI was divided by the total number of fixations in that bin. Participants with more than 25% track loss across all trials and trials with more than 50% track loss were excluded from the analysis.

##### 3.1.4.2 Preprocessing of recall videos

To determine the proportion of correct recall for each participant, we compared the video presented on the eye tracker with the video recorded by the camera. Each video was segmented into subactions based on the changes in the path trajectory. These subactions were identified by counting the inflection points in the path direction, that is, the number of times the ball changes its direction in an entire video. This segmentation resulted in a total of six subactions for actions (a) and (b) (Fig. 2a,b), and eight subactions for actions (c) and (d) (Fig. 2c,d). A correct recall of the subaction was identified when the participants correctly followed the specified path direction: over or under. Conversely, if a participant performed a substitutive subaction, an entirely different subaction, or omitted the subaction, it was classified as incorrect. Each subaction was scored 1 for correct recall and 0 for incorrect recall. Based on the transcription, we calculated the types of errors and its percentage distribution and found that omission errors occurred most frequently (51.82%), followed by substitution errors (24.82%), and other errors (23.36%). Overall, the coding of participants' subactions yielded 640 data points, among which 137 were classified as errors, resulting in an overall accuracy of 78.59%.^2^ The total number of correct subactions were added for each video. The proportion of correct recall was calculated by normalizing the correct subactions by total subactions. For the analysis, a logistic regression model was fitted using a generalized linear mixed effects model (glmer) in R (Barr, 2008). To ensure the reliability of the coding, two student assistants coded the data for which the inter‐rater reliability test was conducted (Kendall's *W* = 0.78).

## 4 Results

### 4.1 Eye movements

**Fig. 3:**
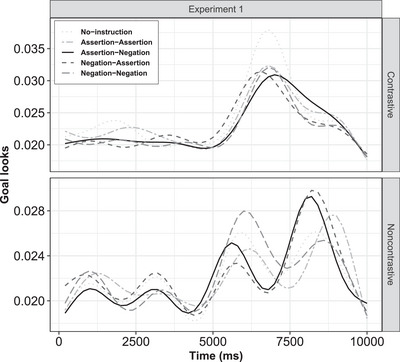
Experiment 1: Estimated goal looks in contrastive (top plot) and noncontrastive action (bottom plot) conditions.

We analyzed the time‐dependent changes to the fixation on the goal object to investigate the attention to the goal. The raw data followed a nonlinear curve, hence we used the Generalized Additive Model (GAM) to model the fixations. GAM has been shown to capture the nonlinearity by penalizing the overfitting and has previously been used to model data in visual world paradigms (Porretta, Kyröläinen, Van Rij, & Järvikivi, 2018; Porretta, Tucker, & Järvikivi, 2016; Wieling, 2018). To evaluate the assumption of nonlinearity, we initially fitted the data using both linear and nonlinear models by incorporating a smoothed (nonlinear) term. An Akaike Information Criterion (AIC) test revealed that the nonlinear model had a lower AIC score compared to the linear model (AIC difference = –8962.10), supporting the justification for nonlinearity. For our analysis, we fitted a separate GAM model for each action type. The fixations were transformed into empirical‐logit to treat it as a continuous response variable (Barr, 2008). The model included parametric terms for instructions, which were treatment contrast‐coded with the no‐instruction condition as the baseline, along with its factor‐smooth interaction with the time bin. A log‐likelihood model comparison was done between a reduced and a full model to estimate the best fitting model following Barr, Levy, Scheepers, and Tily (2013). The Chi‐Square test on the Maximum Likelihood (ML) scores indicated that model with smooth term was significantly better than the null model for both contrastive ($\chi^{2}$(8.00) = 251.31, *p* < .001) and noncontrastive actions ($\chi^{2}$(8.00) = 579.66, *p* < .001). The model summary is provided in Table 1, and the estimated effects at the probability scale are visualized in Fig. 3.

**Table 1:** Experiment 1: Estimates of looks toward the goal for contrastive and noncontrastive actions

Contrastive action			
Parametric coefficients	Estimate	*SE*	*p*
Intercept	−3.71	.003	< .001
Assertive–Assertive	−.02	.004	< .001
Assertive–Negative	−.05	.004	< .001
Negative–Assertive	−.04	.004	< .001
Negative–Negative	−.06	.004	< .001

Smooth terms	edf	*F*	*p*
s(bin):No‐instruction	8.94	358.93	< .001
s(bin):Assertive–Assertive	8.88	221.33	< .001
s(bin):Assertive–Negative	8.72	226.08	< .001
s(bin):Negative–Assertive	8.84	204.24	< .001
s(bin):Negative–Negative	8.91	215.81	< .001

**Noncontrastive action**			
Parametric coefficients	Estimate	*SE*	*p*
Intercept	−3.79	.002	< .001
Assertive–Assertive	.003	.004	.42
Assertive–Negative	.001	.004	.89
Negative–Assertive	.012	.004	.002
Negative–Negative	.02	.004	< .001

Smooth terms	edf	*F*	*p*
s(bin):No‐instruction	8.95	146.01	< .001
s(bin):Assertive–Assertive	8.93	123.92	< .001
s(bin):Assertive–Negative	8.96	191.34	< .001
s(bin):Negative–Assertive	8.95	181.13	< .001
s(bin):Negative–Negative	8.94	174.47	< .001

Abbreviation: Intercept, no‐instruction condition.

(A) Contrastive action: Compared to the baseline (no instruction), the parametric term (time‐independent) revealed a significant main effect of instruction. The estimates indicated that fixation on the goal significantly decreased from the baseline as soon as instructions were provided across all instruction conditions: negative–negative (β = –.06, *p* < .001), assertive–negative (β = –.05, *p* < .001), negative–assertive (β = –.04, *p* < .001), and assertive–assertive (β = –.02, *p* < .001) instructions. Crucially, the time‐dependent smooth terms revealed distinct temporal trends across all the instruction conditions. The assertive–negative condition exhibited the lowest effective degrees of freedom (edf = 8.72, *p* < .001), indicating a smoother trajectory over time compared to the other conditions (Fig. 3, top). To examine this time‐dependent reduction in goal‐looks, we conducted GAM difference smooth comparisons for all the instruction conditions against the baseline (no instruction). As revealed in the visualization, fixation on the goal in the assertive–negative condition significantly decreased in the later time period relative to the baseline.^3^

(B) Noncontrastive action: For the parametric term (time‐independent), a significant difference in goal‐looks from the baseline (no‐instruction) was observed when a negative instruction was provided first, with the higher fixation from the baseline for the negative–negative instruction (β = .02, *p* < .001) and then negative–assertive (β = .012, *p* = .002). Regarding the time‐dependent changes, all the instruction conditions were significantly different from reference, with the lowest edf score for the curve of assertive–assertive condition (edf = 8.93, *p* < .001) (Fig. 3, bottom). To test for the time‐related changes to the goal for each instruction condition from the baseline, we conducted a difference smooth comparison test. For the later time period, we observed a decrease in the goal‐looks for assertive–assertive condition.

### 4.2 Recall

Our aim was to analyze the proportion of correctly performed actions in response to different instructions. A generalized linear mixed effects model was fitted for each action type using glmer function in R with a binomial family (Bates, Mächler, Bolker, & Walker, 2015). The model included the factor instructions, coded using treatment‐contrast ensuring that the intercept represented no‐instruction condition (baseline) against which all instruction conditions were compared. Additionally, the model incorporated a full‐random effect structure for the items and the subjects to account for potential variation arising due to complexity of items and individual differences. The random effects were added stepwise in the model following log‐likelihood test to determine whether their inclusion significantly improved the model fit. The model revealed a between subjects (*SD* = 0.42) and items variance (*SD* = 0.16).^4^

**Fig. 4:**
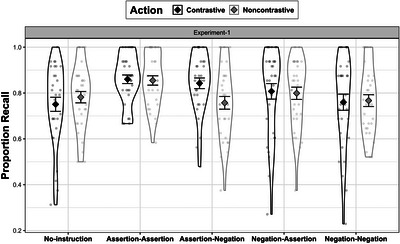
Experiment 1: Proportion of correct recall for contrastive and noncontrastive actions. Instruction conditions are plotted against x‐axis.

A post‐hoc individual pairwise comparison was conducted using the emmeans package to assess the main effect of instruction for each of the actions (Lenth, 2017). The model summary is provided in Table 2, and the results are visualized in Fig. 4. The model revealed that assertive–assertive instruction significantly improved recall for both kinds of actions as compared to the no‐instruction (baseline) condition, suggesting that when actions are combined with assertive instruction, remembering the sequence is enhanced as shown by the positive estimates for noncontrastive (β = .49, 95% CI [.14, .84], *p* = .01) and contrastive (β = .78, 95% CI [.43, 1.14], *p* < .001) actions. Crucially, the recall for the contrastive action also improved significantly from the baseline in the presence of the assertive–negative (contrastive) instruction (β = .64, 95% CI [.29, .98], *p* < .001). A similar pattern was also observed for the negative–assertive condition, another form of contrast, which also showed a positive effect on recall for contrastive actions (β = .42, 95% CI [.09, .75], *p* = .01). In summary, whereas participants showed an overall better recall for both types of action in the presence of assertive instructions, the assertive–negative and negative–assertive (contrastive instructions) specifically facilitated recall for the contrastive actions. Following this result, we tested whether assertive–negative was more effective than only assertive instructions specifically for contrastive actions by doing a pairwise comparison test. The test revealed no significant difference, which indicates that the recall of a contrastive action under the assertive–negative (contrastive) was as good under the assertive–assertive instruction. For noncontrastive actions, only assertive instructions significantly improved action recall, suggesting that participants were more likely to score better on memory task when accompanied by only assertive (noncontrastive) instructions and that using negation for such actions was perhaps implausible. In contrast, for contrastive actions, recall was enhanced when paired with either assertive–negative (contrastive) or assertive–assertive (noncontrastive) instruction.

**Table 2:** Experiment 1: Parameter estimates for the recall for contrastive and noncontrastive actions for levels of verbal instructions

Noncontrastive action	Estimate	SE	95% CI	*p*
Intercept	1.41	.29	[.83, 1.98]	< .001
Assertive–Assertive	.49	.18	[.14, .84]	.01
Assertive–Negative	−.17	.16	[−.49, .15]	.29
Negative–Assertive	.07	.17	[−.26, .40]	.67
Negative–Negative	−.08	.16	[−.40, .24]	.61

**Contrastive action**	Estimate	*SE*	95% CI	*p*
Intercept	1.28	.38	[.54, 2.02]	< .001
Assertive–Assertive	.78	.18	[.43, 1.14]	< .001
Assertive–Negative	.64	.18	[.29, .98]	< .001
Negative–Assertive	.42	.17	[.09, .75]	.01
Negative–Negative	.07	.16	[−.25, .39]	.68

Abbreviations: CI, confidence interval; Intercept, no‐instruction condition.

## 5 Discussion: Experiment 1

In our first experiment, we investigated the effect of verbal guidance on the attention to the goal and action recall. The verbal guidance consisted of verbal instructions, for which we used requests containing negation versus assertion, and combined them with the action demonstration for the first time. In this setup, the instructions were presented before the actions, and the offset of the instruction marked the beginning of an action (Fig. 1a). Our primary focus was on understanding how the time‐dependent changes in the attention toward the goal shaped the action memory. The findings suggest that verbal instructions play a pivotal role in modulating the attention on the action goal. This is shown by a notable reduction in attention to the goal when participants received an assertive–negative instruction before a contrastive action.

In a contrastive action, the positive and its negative counterpart mapped onto a single semantics, which might have potentially brought the two paths following each other into a unified frame. In our experiment, the latter path was the alternate of the prior path, thus, the use of negation had two roles to play at the goal final object: inhibit the current subaction and highlight the absence of the prior subaction. The process of inhibition and reference to the previous subaction together might have contributed to the reduction in the goal‐looks at the later time. Although guiding a contrastive action with a negation first (e.g., NOT Under–Now Under) also reduced the attention to the goal to some extent (see Fig. 3, top), this attenuation is higher when the negation is used for the later subaction (Now Over–NOT Over). This highlights the benefits of a prior context (assertive) before the negation can be used (Albu et al., 2021; Kaup et al., 2006; Wason, 1965).

For the noncontrastive action, reduction in the attention to the goal was noticeable for the assertive guidance at later time interval. In this case, the presence of an assertive guidance during the later subaction served to reiterate the preceding subaction.

Importantly, the modulation of attention from the goal affected the action memory. For contrastive context, we found that the memory was significantly enhanced when a negation was used at the later subaction (e.g., Now Over–NOT Over). This phenomenon can be attributed to the contextual processing of negation (Wason, 1965), wherein negation appears to be more informative by integrating prior and current subevents within a unified conceptual framework. Crucially, according to the two‐steps processing account, it has been suggested that when the positive counterpart is already under consideration, the activation of the alternate does not necessarily occur upon encountering negation (Kaup et al., 2007; Kaup & Dudschig, 2020). This alleviates processing costs and facilitates negation processing. Presenting the assertive instruction at the earlier subaction and negating the same action later—particularly in a context where the negated current subaction was previously affirmed—may have resulted in a single‐step processing.

Additionally, we observed that assertive instructions enhanced the memory for both types of actions in general, which aligns with the previous findings that a multimodal presentation of an action enhances its perception (Brand & Tapscott, 2007; Hirsh‐Pasek & Golinkoff, 1996; Rohlfing, Fritsch, Wrede, & Jungmann, 2006; Sciutti et al., 2016; Wrede, Schillingmann, & Rohlfing, 2013). Importantly, this perception has also been shown to be sensitive of how the modalities concerned are aligned (Bahrick & Lickliter, 2000, 2002; Bahrick et al., 2004). Although for a contrastive action, we observed a more reduction in the attention to the goal and better action memory in the presence of an assertive–negative instruction, this condition did not differ from the simple assertive instruction. One possible explanation is that not all participants might have benefited equally from negation, and individual differences in processing negation could have weakened the overall effect, especially given that negation is generally more cognitively demanding than its affirmative counterpart (Clark & Chase, 1972; Kaup & Dudschig, 2020).

The way in which verbal instructions can meddle with the action demonstration can be complex (Wolff & Holmes, 2011). Whereas in the first experiment, we explored the possibility that verbal instructions presented before actions could spotlight the upcoming action, another possibility is to offer language simultaneously to better match it with the ongoing actions. Since action perception is shown to be sensitive to the alignment of the modalities, whether sequential (Nomikou & Rohlfing, 2011; Sciutti et al., 2016), or simultaneous (Bahrick & Lickliter, 2000, 2002), we addressed the role of multimodal synchrony in a follow‐up experiment. Particularly, in the second experiment, we aimed to investigate what consequence a different form of instruction‐action synchrony has on the ever‐present goal bias and action perception, such as when the instruction overlaps with the action.

## 6 Experiment 2: Instruction along with the action

In our follow‐up experiment, we extended the approach of first experiment by introducing the verbal instruction along with actions, such that the instructions overlapped with the actions (Fig. 1b). In a study with infants, it was found that the timing of verbal instruction plays a crucial role in highlighting the subactions. For example, mothers introduced the verbal instruction before a subaction to direct attention and highlight it more than other components (Nomikou & Rohlfing, 2011; Sciutti et al., 2016). Conversely, studies also suggest that verbal instructions overlapping with actions mark distinct boundaries of an action, thus, facilitate the segmentation—a phenomenon known as “acoustic packaging” (Brand & Tapscott, 2007; Hirsh‐Pasek & Golinkoff, 1996; Rohlfing et al., 2006; Wrede et al., 2013). It has been argued that acoustic packaging essentially organizes a perception of meaningful units of actions by chunking them into subactions (Brand & Tapscott, 2007; Rohlfing et al., 2006; Schillingmann, Wrede, & Rohlfing, 2009; Wrede et al., 2013). Furthermore, presenting instructions overlapping with the subaction leads to a unitary perception of the modalities due to intersensory redundancy, a principal for which our cognition is developmentally shaped and generally offers an advantage for early learning (Bahrick & Lickliter, 2000, 2002; Bahrick et al., 2004). For such unitary patterns, the default behavior of our perceptual system is to prioritize the action goal, a tendency that also appears to be socially learned for most events due to sensorimotor processes (Lakusta & Landau, 2012; Wolff & Holmes, 2011). Thus, due to the instruction‐action overlap in Experiment 2, we expected to observe greater attention to the action goal compared to Experiment 1.

## 7 Method

### 7.1 Participants

Forty‐two university students (mean age = 24.45, *SD* = 3.2) participated in the experiment. All the participants were native or fluent German speakers. An informed consent was obtained from all the participants prior to data collection following the university ethical guidelines. Data from five participant were excluded from the analysis due to the track‐loss for more than 50% of the trial or for failing to properly follow the instruction. Thus, the final sample size for the experiment was 37, which was comparable to the first experiment.

### 7.2 Stimuli

The stimuli were the same as those used in the first experiment, except that the verbal instructions now overlapped with the actions.

### 7.3 Procedure

Participants followed the same procedure and were briefed about the experiment in the same way as in the first experiment. Similarly, all the experiment sessions started with 10 practice trials, where participants familiarize themselves with the experiment setup and the objects.

### 7.4 Data processing

For all our analyses, we followed the procedure similar to our first experiment. This included using the same statistical models and coding schema to retain the uniformity across two experiments.

#### 7.4.1 Eye movements preprocessing

Similar to the first experiment, we created a polygon AOI around the goal object. To retain the consistency of the AOI, we imported the AOI presets from our first experiment.

#### 7.4.2 Preprocessing of recall videos

The recall videos from video cameras were compared with the videos shown on the eye tracker screen and coded in binary, 1 for correct and 0 for incorrect recall. The coding was performed by two student assistants providing the inter‐rater reliability (Kendall's *W* = 0.84). Following this, we calculated the total error percentage and its distribution across error types. The coding yielded a total of 720 data points, with 130 classified as errors, resulting in an accuracy rate of 81.94%. Among the error types, omission errors were the most frequent (53.08%), followed by other errors (26.92%) and substitution errors (20%).^5^

## 8 Results

### 8.1 Eye movements

**Fig. 5:**
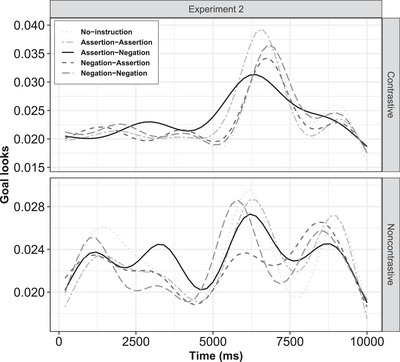
Experiment 2: Estimated goal‐looks for noncontrastive action (top plot) and contrastive action (bottom plot).

Following the analysis from our first experiment, we used GAM to fit the fixation data since the fixation pattern followed a nonlinear trend. The response variable was empirical‐logit transformed prior to fitting the model following Barr (2008). We were specifically interested in examining the temporal pattern of attention directed toward the goal throughout the trial. A separate GAM model was fitted for each action type by incorporating the instruction as the parametric component and its factor smooth interaction by time. The predictor variables were treatment contrast‐coded such that the model intercept represented the baseline condition (no‐instruction). To select the best‐fitting model, a log‐likelihood model comparison was done between a reduced and full model following Barr et al. (2013). The Chi‐Square test on the Maximum Likelihood (ML) scores indicated that model with instruction‐time interaction was significantly better than the null model without interaction term, for contrastive ($\chi^{2}$(8.00) = 751.35, *p* < .001) and noncontrastive action ($\chi^{2}$(8.00) = 648.48, *p* < .001). The model summary is provided in Table 3, and the estimated effects are visualized in Fig. 5, respectively.

**Table 3:** Experiment 2: Estimates of looks toward the goal for contrastive and noncontrastive actions

**Contrastive action**			
Parametric coefficients	Estimate	*SE*	*p*
Intercept	−3.75	.003	< .001
Assertive–Assertive	.016	.004	< .001
Assertive–Negative	−.037	.004	< .001
Negative–Assertive	−.006	.004	.18
Negative–Negative	.019	.004	< .001

Smooth terms	edf	*F*	*p*
s(bin):No‐instruction	8.98	364.68	< .001
s(bin):Assertive–Assertive	8.97	475.49	< .001
s(bin):Assertive–Negative	8.76	215.45	< .001
s(bin):Negative–Assertive	8.95	280.78	< .001
s(bin):Negative–Negative	8.96	364.45	< .001

**Noncontrastive action**			
Parametric coefficients	Estimate	*SE*	*p*
Intercept	−3.74	0.003	< .001
Assertive–Assertive	−.013	.004	< .001
Assertive–Negative	.003	.004	.46
Negative–Assertive	−.034	.004	< .001
Negative–Negative	−.024	.004	< .001

Smooth terms	edf	*F*	*p*
s(bin):No‐instruction	8.96	204.13	< .001
s(bin):Assertive–Assertive	8.95	172.11	< .001
s(bin):Assertive–Negative	8.91	77.72	< .001
s(bin):Negative–Assertive	8.86	95.91	< .001
s(bin):Negative–Negative	8.95	151.08	< .001

Abbreviation: Intercept, no‐instruction condition.

(A) Contrastive action: Similar to the first experiment, when instruction conditions were compared to the baseline (no‐instruction), the parametric terms (time‐independent) revealed a significant effect for the instructions, such that attention to the goal significantly decreased for assertive–negative condition (β = –.037, *p* < .001). Crucially, the assertive–negative instruction was contrastive, replicating our previous results from the first experiment. Moreover, the effects observed were more pronounced than in the previous experiment (Fig. 5, top). In contrast, other instruction conditions, such as assertive–assertive and negative–negative, led to a significant increase in goal‐looks from the baseline, as shown by the positive estimates for assertive–assertive (β = .016, *p* < .001) and negative–negative (β = .019, *p* < .001) instruction conditions.

Following this, we examined the time‐dependent terms and found that all smooth terms were significantly different from the reference level, indicating distinct temporal trends across conditions. Notably, as in the first experiment, the assertive–negative condition showed the lowest effective degrees of freedom (edf = 8.76, *p* < .001), with its effect emerging earlier than in Experiment 1. To further investigate this, we additionally conducted a GAM difference smooth comparison for each instruction condition against the baseline (no‐instruction). Consistent with the first experiment, the difference plot revealed that assertive–negative condition led to a reduction in goal‐looks, followed by the assertive–assertive condition.^6^

(B) Noncontrastive action: For the parametric (time‐independent) component, there was a significant difference in the goal‐looks between baseline (no‐instruction) and other instruction conditions, such that fixation to goal significantly decreased for all the instruction conditions except when assertive–negative instruction was provided (β = .003, *p* = .46). For the time‐dependent smooth terms, we observed significant different temporal pattern for all the instruction conditions as compared to the reference level. Although we did not find a lower edf score for the assertive–assertive instruction, as was observed in Experiment 1, a GAM difference smooth comparison revealed that fixation on the goal decreased significantly in both the assertive–assertive and assertive–negative conditions compared to the baseline.

Overall, our finding for the contrastive action replicated our results from the first experiment. We found reduced attention to the goal, compared to the baseline, when a contrastive action was paired with an assertive–negative (contrastive) instruction, as shown by the smooth difference comparisons. For the noncontrastive actions, in Experiment 2, we observed a decrease in goal‐looks for both assertive–assertive and assertive–negative conditions compared to the baseline (no‐instruction). While the effect for the assertive–assertive condition replicated the findings from the first experiment, the decrease in attention to the goal in the assertive–negative condition in the second experiment was unexpected and might reflect the benefit of using an assertion at the first subevent for noncontrastive actions. Notably, the peaks in the goal‐looks occurred earlier in this experiment than in the first experiment, and the effect was more pronounced. While the influence of multimodal overlapping on attention was already visible here, turning back to our hypothesis on instruction‐action temporal synchrony, we analyzed early and late goal‐look behaviors separately by statistically comparing the two experiments (see Section 9.3).

### 8.2 Recall

**Fig. 6:**
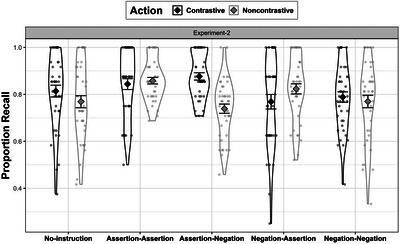
Experiment 2: Proportion of correct recall for contrastive and noncontrastive actions. Instruction conditions are plotted against x‐axis.

For the analysis of the recall data, we used our earlier generalized linear mixed model tofit the complete dataset across both experiments, and conducted a log‐likelihood test between the full and reduced models (Barr et al., 2013). When compared across experiments, we did not observe a significant increase in the score for the second experiment (instruction along with the action), suggesting that the recall remains invariant to the instruction‐action synchrony. Following this, we tested the main effect of instruction on action recall focusing specifically on Experiment 2. The model included the main predictors by treatment‐coding, such that the intercept represented the no‐instruction (baseline) condition. To account for the variances arising due to the items or the subjects, both were treated as the random factors. Adding the by‐item adjustment to the intercept, however, did not significantly improve the model. A post‐hoc multiple comparison was conducted using the emmeans package to obtain the estimates for the factors of interest after the model fitting. The model summary is provided in Table 4, and the estimates are visualized in Fig. 6.

**Table 4:** Experiment 2: Parameter estimates for the recall for contrastive and noncontrastive actions for levels of verbal instructions

Noncontrastive action	Estimate	SE	95% CI	*p*
Intercept	1.33	.15	[1.03, 1.63]	< .001
Assertive–Assertive	.63	.16	[.32, .95]	< .001
Assertive–Negative	−.15	.14	[−.43, .14]	.31
Negative–Assertive	.33	.15	[.03, .62]	.03
Negative–Negative	.00	.15	[−.29, .29]	1.00

**Contrastive action**	Estimate	*SE*	95% CI	*p*
Intercept	1.64	.20	[1.24, 2.04]	< .001
Assertive–Assertive	.36	.20	[−.03, .76]	.07
Assertive–Negative	.55	.17	[.22, .89]	< .001
Negative–Assertive	−.16	.19	[−.52, .20]	.39
Negative–Negative	−.17	.15	[−.47, .13]	.28

Abbreviations: CI, confidence interval; Intercept, no‐instruction condition.

Beginning with the effect of assertive instruction when compared against baseline, we found that an assertive–assertive instruction led to a significant increase in recall only for the noncontrastive action (β = .63, 95% CI [.32, .95], *p* < .001) but not for the contrastive action (β = .36, 95% CI [–.03, .76], *p* = .07). This pattern was different from the first experiment (where the instruction preceded the action), in which assertive instruction facilitated memory for both types of actions. Conversely, in Experiment 2, we observed that an assertive–assertive (noncontrastive) instruction particularly facilitating recall for the noncontrastive action (see Table 4). For the contrastive instruction, we observed that assertive–negative instruction significantly improved the recall for the contrastive action, as indicated by a positive estimate (β = .55, CI [.22, .89], *p* < .001). This result was consistent with the findings from the first experiment, where assertive–negative (contrastive) instructions were shown to be beneficial only for contrastive but not for noncontrastive action. Following this, we tested whether a different form of verbal contrast (negative–assertive) significantly increased the memory for contrastive action as observed in the first experiment. Contrary to the first experiment, in the second experiment, a negative–assertive instruction did not facilitate memory for the contrastive actions. To test whether assertive–negative was significantly better than only assertive instructions for contrastive action, a post‐hoc pairwise comparison was applied using emmeans package (Lenth, 2017). The comparison revealed no significant difference between the assertive–assertive and assertive–negative conditions, suggesting that although recall for contrastive actions was higher under assertive–negative instructions, the improvement was not statistically significant compared to assertive‐only guidance. In summary, when compared to the first experiment, two findings are particularly notable: (a) a verbal contrast in the form of assertive–negative played a crucial role in particularly guiding the contrastive actions, and (b) assertive–assertive instructions selectively enhanced memory for the noncontrastive action—a finding which diverged from the first experiment, where assertive–assertive facilitated memory for both types of actions, irrespective of whether it was contrastive or noncontrastive.

### 8.3 Effect of instruction–action synchrony on attention to the goal

To evaluate the effect of instruction synchrony on the attention to the goal, we analyzed the data across two experiments. Our predictions were derived from two rationales: Whereas in Experiment 1, we explored the possibility that presenting the instruction as a spotlight before the subaction might interfere with early sensorimotor processes, in Experiment 2, we expected that the overlapping presentation of the modalities would lead to a unitary perception—thereby naturally directing attention toward the action goal as a result of automatized sensorimotor routines (Bahrick & Lickliter, 2000; Bahrick et al., 2004; Zacks & Swallow, 2007). Accordingly, we predicted that attention to the action goal would be higher in Experiment 2 compared to Experiment 1.

**Fig. 7:**
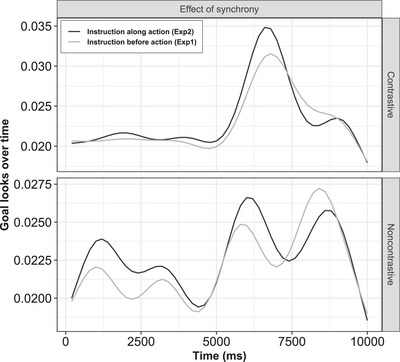
Effect of forms of synchrony on goal‐looks over time for contrastive and noncontrastive actions. The error‐band represents 95% CI.

For the analysis, we only included the trials, where verbal instructions were provided by excluding the baseline (no‐instruction) trials, since the effect of synchrony would only be present when there is an instruction. The data was fitted on the previous GAM model by including experiment number as the main predictor. To observe a temporal difference in attention to the goal across two forms of synchrony, a time‐dependent test of significance was done using mgcv package in R (Wood, 2011). The overall difference in the attention toward the goal across two experiments is shown in Fig. 7, and the estimates of comparisons are shown in Table 5. The parametric terms suggested that when instruction overlapped with the action (instruction along action), it resulted in significantly higher looks to the goal, as shown by positive estimates for both contrastive (β = .02, *p* < .001) and noncontrastive actions (β = .03, *p* < .001). Conversely, presenting instructions before the action reduced attention to the goal (Fig. 7). We also observed that the effect of synchrony on looks to the goal emerged earlier for the noncontrastive than contrastive actions. Given the limited studies investigating oculomotor behavior in contrastive motion events, we refrained from making a concrete prediction on this aspect. However, previous research has suggested that path complexity influences eye movements, such that complex paths are associated with greater cognitive load, leading to more frequent regressions and increased fixation durations (Kosch, Hassib, Woźniak, Buschek, & Alt, 2018). Based on this, we suggest that the earlier fixation on the goal in the noncontrastive condition might be due to the relative simplicity of the unidirectional paths, compared to the bidirectional nature of contrastive paths. That said, it remains unclear whether changes in direction were actually perceived as complex within the flow of continuous motion events, a question that remains open for future investigation.

**Table 5:** Comparison between first and second experiment for the effect of synchrony on attention to the goal

**Contrastive action**			
Parametric coefficients	Estimate	*SE*	*p*
Intercept	−3.76	.01	< .001
Instruction along action	.02	.002	< .001

Smooth terms	edf	*F*	*p*
s(bin):Instruction before action	8.95	811.40	< .001
s(bin):Instruction along action	8.99	1246.30	< .001

**Noncontrastive action**			
Parametric coefficients	Estimate	*SE*	*p*
Intercept	−3.79	.001	< .001
Instruction along action	.03	.002	< .001

Smooth terms	edf	*F*	*p*
s(bin):Instruction before action	8.97	435.30	< .001
s(bin):Instruction along action	8.98	438.10	< .001

### 8.4 Does attention to the goal influence action recall?

Whereas the multimodal synchrony affected the attention to the goal, we conducted an analysis to investigate whether it relates to the action memory. Specifically, we examined the relationship between the attention to the goal and action memory. The aim was to test whether the recall scores negatively predict the overall looks toward the goal. This will inform us that whether high attention to the goal leads to a coarse perception of intermediate events, ultimately shaping the overall memory of the action. Thus, the data from two experiments was modeled together by treating the recall score as the predictor and fixation on the action goal as an outcome variable. To preserve the time dimension, we followed a method previously used to analyze visual‐world data (Porretta et al., 2018; Porretta et al., 2016; Wieling, 2018). The recall score was mean‐centered for better convergence. To account for variability in looking patterns between subjects and items, a random intercept adjustment to the subject and item was added to the model. This decision was based on a “goodness of fit” test comparing models with and without random adjustments. The random effects structure captures differences such as whether certain participants are early or late in looking at the goal, and whether specific items influence distinct looking patterns. We expected that the recall score would negatively predict the fixation on the goal if there exists an inverse relation between the action memory and the attention to the goal. The model comparison was done using log‐likelihood method, which indicated that including the recall score in the model led to a significant better fit ($\chi^{2}$(8.00) = 1244.67, *p* < .001).

**Fig. 8:**
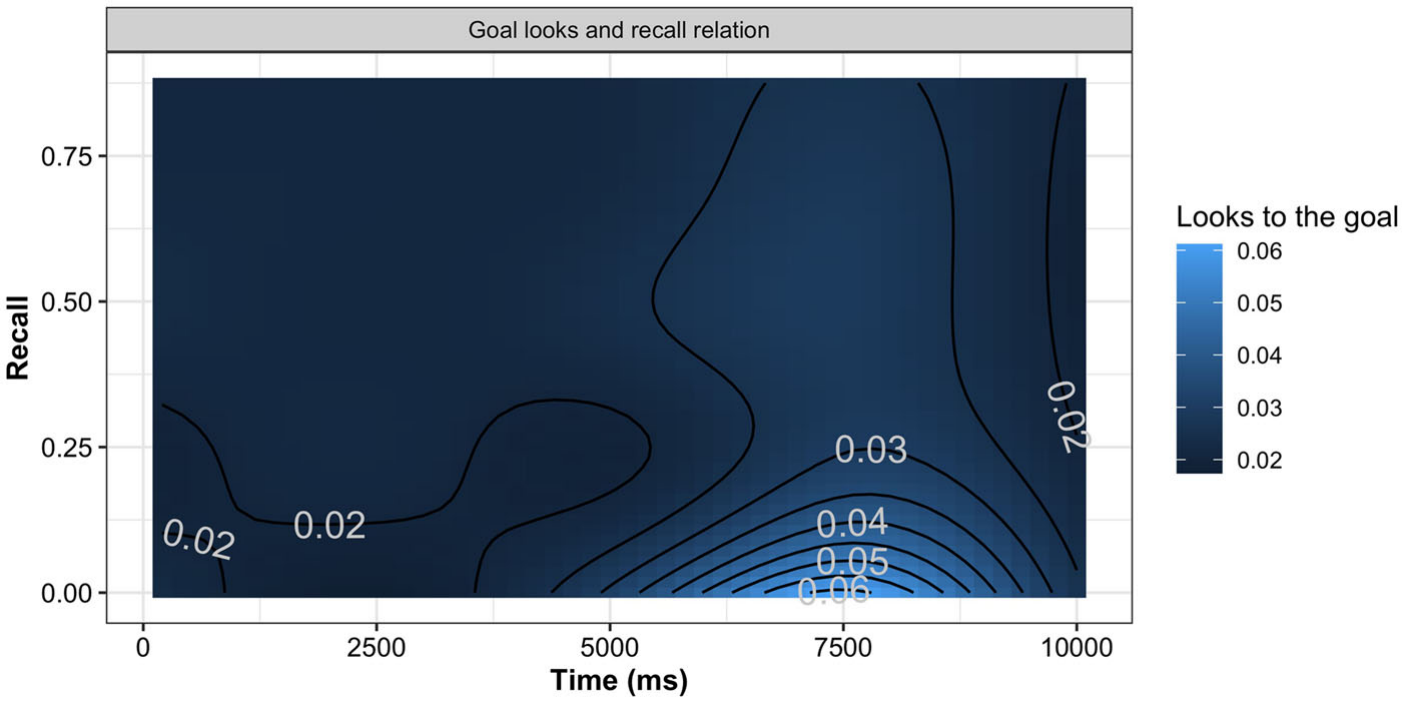
Plot for the attention to the goal and action recall. Y‐axis shows the recall score and x‐axis shows the time from beginning of the trial. The numbers indicate the probability of looks toward the goal with white shading an increase in the goal‐looks.

The predictions are shown in Table 6, and the results are visualized in Fig. 8. There was a significant negative effect of score such that the recall score negatively predicted the looks toward the goal (β = –.26, 95% CI [–.32, –.17], *p* < .001). To put it in other words, participants' recall was better when they managed to “de‐bias” themselves from the goal. The effect predominantly occurred approximately between 5000 and 9000 ms window peaking at approximately 7500 ms. This was also the peak time for the looks toward the goal across two experiments as observed for different forms of modalities alignment (Fig. 7). Additionally, the model revealed a variation between subjects (*SD* = 1.40) and items (*SD* = 0.31). ^7^

**Table 6:** Parameter estimates for the recall score and fixation

Terms	Estimate	*SE*	95% CI	*p*
Intercept	−4.87	.23	[−5.32, 4.42]	< .001
c_mean_recall	−.26	.04	[−.32, −.17]	< .001

## 9 Discussion: Experiment 2

The aim of the second experiment was to address other possibilities of language interplaying with the actions. In the first experiment, the instruction was presented before the action, in the second experiment, we presented the instruction along with the action to “package” it by overlapping the modalities (Brand & Tapscott, 2007; Hirsh‐Pasek & Golinkoff, 1996; Schillingmann et al., 2009). We introduced the overlapping to validate our findings for the goal attention and action memory across two forms of instruction–action synchrony. With overlapping modalities, we observed that the verbal contrast (assertive–negative) led to a more pronounced reduction in the attention to the goal for the contrastive action. The co‐occurrence of the instruction and action has been proposed to enhance one's ability to see the sequence of actions as unitary, a phenomenon rooted in developmental processes and considered relevant for action learning (Bahrick et al., 2004; Brand & Tapscott, 2007). It is also worth noting that interpreting negations has always been difficult than assertions (Carpenter & Just, 1975; Clark & Chase, 1972), also in the context of actions (de Vega et al., 2016; Tettamanti et al., 2008). Hence, overlapping the negation with an action might have made the path of an action easier to interpret as opposed to when negation is presented before the action. Thus, the verbal guidance comprising negation is more sensitive to the multimodal overlap as compared to an assertive guidance. This was particularly observable for the recall. In the second experiment, we observed a recall pattern similar to that of the first experiment, with two notable differences: (a) Whereas in the first experiment, assertive instructions facilitated recall for both contrastive and noncontrastive actions, in the second experiment, assertive instructions selectively enhanced memory for noncontrastive actions, and similarly, (b) whereas recall for contrastive actions in the first experiment was facilitated by both forms of verbal contrast (assertive–negative or negative–assertive), in the second experiment, only assertive–negative instruction significantly improved recall for contrastive actions. Thus, compared to Experiment 1, in Experiment 2, when the instruction overlapped with the action, the role of verbal instruction became more tailored to the specific type of action it guided.

## 10 General discussion

To enhance the demonstration of actions, verbal instructions are used. They can contain assertive or negative information. Whereas many studies reveal the effect of assertion versus negation processing, an embededdness of this processing in the context of an action demonstration is lacking but could be insightful for the setting of joint work and learning.

For our study, we linked together research from action demonstration with linguistic research on negation to offer effective verbal guidance. We conducted two eye tracking experiments where the verbal instructions were presented either before the action (Experiment 1) or along with the action (Experiment 2). This was done to foster two forms of synchrony between instruction and action, in line with the theory of “acoustic packaging”(Brand & Tapscott, 2007, p. 323) and intersensory redundancy (Bahrick et al., 2004). Crucially, the instructions were a sequence of assertion and negation, combined with action presentation that was either contrastive or noncontrastive. To remind the reader, with a contrastive action, we refer to two subactions that contrast with each other (e.g., OVER–UNDER), while with noncontrastive action, we refer to two recurrent subactions (e.g., OVER–OVER).

Our findings shed light on how the timing and the nature of instruction influence the attention and action memory. We observed that contrastive instructions (assertive–negative) attenuated the attention from the goal for contrastive actions. This pattern was visible in both forms of instruction–action synchrony, regardless of whether the instructions were presented before or along with the action.

When comparing across the experiments, we found that overlapping the modalities led to an increased attention on the goal, compared to when the instructions were presented right before the action (Fig. 7). This was in line with our predictions regarding instruction–action synchrony (Fig. 1), which was grounded in the Intersensory Redundancy Hypothesis (IRH) (Bahrick & Lickliter, 2000, 2002; Bahrick et al., 2004). According to IRH, the co‐occurrence of modalities leads to a reduced selective attention on any one modality, resulting in a unitary perception (Bahrick et al., 2004). This unitary perception plays an ecological role in early learning by supporting the integration of multimodal properties, which lays the foundation for perceptual processing in adulthood. Since these properties are foundational and sometimes considered to be a part of sensorimotor routines (Zacks et al., 2001), the default behavior of our perceptual system is to prioritize action goal in such situations, unless attention is ostensively directed toward a specific subaction, through language spotlighting (Wolff & Holmes, 2011). This was the case when instructions were presented before the action. In this context, the instructions rather served to ostensively introduce and highlight a particular subaction to which it was intended to guide. Thus, presenting instruction before the subaction might ensure sustained attention to that particular subaction due to selective attention on each modality at a given time (here: verbal or visual). Given that there has been a trade‐off between goal‐attention and the intermediary components (Ikegami, 1979; Papafragou, 2010; Zacks & Swallow, 2007), the overlapping condition in Experiment 2 may have resulted in reduced selective attention to either modality, manifesting as an inherent tendency, such as increased looks toward the action goal. Thus, the action–instruction synchrony involves a delicate balance between facilitating attention to the goal and subactions, which might serve different purposes for communication. To highlight an immediate subaction, providing an instruction before the action can enhance sustained attention on that particular subaction. Conversely, if the purpose is to ensure more attention on the goal than the mid‐events, presenting instructions along with the action can be more beneficial. This is the case for children, who certainly need to discern the relevance of the action for a specific goal, and thus, it is not surprising that action–verbal instruction synchrony was found to be high with younger children (Nomikou & Rohlfing, 2011). For event cognition research, it is important to emphasize that experiments investigating online attention to the goal during event conceptualization should carefully take the timing of instruction in consideration. This is particularly crucial, as attention to the goal appears to be sensitive to instruction–action synchrony.

Our study further highlights that the contrastive instruction differs from mere assertive instruction due to the role of negation in generating a rich contextual effect that is appropriate for a contrastive action. In a contrastive action, the later subaction was the opposite of the prior subaction. Thus, negating the later subaction entails referring to the previous subaction. This unfolding context at verbal and visual level might have led to an easier interpretation of the whole action. The interpretation aligns with the premise that contrastiveness relies on connecting later subevent with the preceding ones (Talmy, 1985, 1975, 2000), and when the modalities temporally overlap, this connection is more profound (Brand & Tapscott, 2007; Gogate, Bahrick, & Watson, 2000; Hirsh‐Pasek & Golinkoff, 1996; Rohlfing et al., 2006). Furthermore, this also connects with findings in the literature showing that people often use negation to prevent the repetition of a past action and to inhibit a prepotent or biased response (e.g., Beltrán et al., 2021; de Vega et al., 2016). Our results correspond with this, suggesting that contrastive instructions may serve a similar inhibitory function by modulating attention and facilitating memory for contrastive actions. For example, in a teaching scenario, instructors often use negation to achieve these dual objectives—preventing repetitive actions and inhibiting bias responses—due to negation's ability to influence domain‐general inhibitory mechanisms. As an extension, an effective approach for guiding an action should employ a forward referencing strategy in addition to a negation. In this approach, one not only inhibits the response bias by referring back and contrasting with past action(s), but also states what needs to be done in place, as in the example, “Do not move it right, but left!” A recent study has utilized this approach to show that contrasting two opposing manners in an action fosters learning of a targeted manner (Groß et al., 2023). In this context, negation can serve as a powerful linguistic tool for guiding such behaviors by inhibiting expected responses and contrasting them with the past action(s). Crucially, contrastive guidance strategy has the potential to attenuate the attention directed toward the goal that arises from repeated expectations. It would also be intriguing to explore the broader implications of such guidance in real‐world scenarios, investigating whether these effects extend beyond actions and impact memory retention over time. This exploration could have significant implications for learning or understanding actions, particularly in terms of how individuals process and retain attenuated information, such as the manner of an action, in addition to the salient properties, such as path (Papafragou et al., 2008; Papafragou, 2010). In this regard, our findings revealed that attenuated attention from the goal led to a more holistic understanding of an action. This finding suggests a fundamental trade‐off between attention to the goal and event memory: when attention is biased toward the goal, other preceding subevents might be encoded less robustly. This resonates with the previous findings, such as the source–goal asymmetry in event memory (Lakusta & Landau, 2012; Papafragou, 2010), encoding of the path and the manner (Papafragou, 2010; Skordos et al., 2020), and prioritization of the goal over the means (Bird, Brindley, Leighton, & Heyes, 2007). Our study also highlights the potential use of “acoustic packaging” in such scenarios. By packaging the intermediate subactions and highlighting the fine‐grained aspects, acoustic packaging may mitigate the detrimental effects of the potential biases on event memory. Our study was limited by examining the attention particularly on the goal and we did not directly measure the attention on the subactions. Future studies should fill this gap by simultaneously assessing the attention on the subaction(s) and the action goal to observe the real‐time attentional trade‐off between these two constituents in an action, particularly for two forms of multimodal synchrony.

In conclusion, our study demonstrated the robustness of assertive instructions in enhancing the action recall and highlighted the potential of negation to guide contrastive actions. It further suggests a delicate balance between the goal attention and event memory underlying the action perception. In our study, participants' recall was better when they managed to “de‐bias” themselves from the goal. Future studies should delve into the question of whether and how visual and verbal contrasts together shape our memory and everyday perception, particularly when used in an interactional context Supporting Information.

## Supporting information



Table S1: Estimates for post‐hoc pairwise comparison for recall (Experiment 1)
Table S2: Estimates for post‐hoc pairwise comparison for recall (Experiment 2)
Table S3: Experiment 1—Classification of errors made during recall when an incorrect subaction was identified
Table S5: Random effects estimates and plots showing recall by subject and by item for Experiment 1
Table S4: Experiment 2—Classification of errors made during recall when an incorrect subaction was identified
Table S6: Random effects estimates and plots showing recall by subject and by item for Experiment 2
Table S7: Random effects estimates and plots for goal‐looks by subject and by item for action types in Fig. 2: C1 (Action A), C2 (Action B), C3 (Action C), and C4 (Action D).
Figure S3: GAM difference smooth comparison between no‐instruction and other instruction conditions as reported for Figs. 3 and 5 in the paper

## Data Availability

The data and analysis codes that support the findings of this study are openly available in OSF at https://osf.io/zq7b2/?view_only=f571b0c6f306476792f20e963db3ca4c

## Notes

1 Information on sample size calculation is provided in related OSF repository.

2 A table of the error types is provided in the Supplementary Materials.

3 GAM difference plots are provided in the Supplementary Materials.

4 The random effects table and plots are provided in the Supplementary Materials.

5 A full table of each error type is provided in the Supplementary Materials.

6 GAM difference plots are provided in the Supplementary Materials.

7 Estimates and plots for random effect structure are provided in the Supplementary Materials.
