# Supplementary material for: Contrastive Verbal Guidance: A Beneficial Context for Attention To Events and Their Memory?
Source: Cogn Sci. 2025 Aug 14;49(8):e70096. doi: 10.1111/cogs.70096 (PMC12352477; doi:10.1111/cogs.70096)

Supplementary materials

Table 1: Estimates for post hoc pairwise comparison for recall (Experiment 1)

Best model: recall_score ~ instruction*path + (1|Subj) + (1|path)

Simple contrasts for instruction.

Path: Noncontrastive

Contrast	Estimate	SE	*z*.ratio	*p*.value
(No-instruction) - (Assertion-Assertion)	-0.2610	0.148	-1.768	0.3922
(No-instruction) - (Assertion-Negation)	0.1629	0.143	1.142	0.7842
(No-instruction) - (Negation-Assertion)	0.0104	0.144	0.072	1.0000
(No-instruction) - (Negation-Negation)	0.0414	0.144	0.288	0.9985
(Assertion-Assertion) - (Assertion-Negation)	0.4239	0.146	2.901	0.0305
(Assertion-Assertion) - (Negation-Assertion)	0.2714	0.147	1.840	0.3503
(Assertion-Assertion) - (Negation-Negation)	0.3024	0.147	2.055	0.2401
(Assertion-Negation) - (Negation-Assertion)	-0.1525	0.143	-1.070	0.8221
(Assertion-Negation) - (Negation-Negation)	-0.1215	0.142	-0.854	0.9134
(Negation-Assertion) - (Negation-Negation)	0.0310	0.144	0.216	0.9995

Path: Contrastive

Contrast	Estimate	SE	*z*.ratio	*p*.value
(No-instruction) - (Assertion-Assertion)	-0.3998	0.146	-2.748	0.0473
(No-instruction) - (Assertion-Negation)	-0.3886	0.145	-2.673	0.0579
(No-instruction) - (Negation-Assertion)	-0.2359	0.143	-1.647	0.4675
(No-instruction) - (Negation-Negation)	-0.0199	0.141	-0.141	0.9999
(Assertion-Assertion) - (Assertion-Negation)	0.0112	0.150	0.075	1.0000
(Assertion-Assertion) - (Negation-Assertion)	0.1639	0.148	1.109	0.8019
(Assertion-Assertion) - (Negation-Negation)	0.3799	0.146	2.608	0.0689
(Assertion-Negation) - (Negation-Assertion)	0.1526	0.148	1.034	0.8396
(Assertion-Negation) - (Negation-Negation)	0.3686	0.146	2.534	0.0833
(Negation-Assertion) - (Negation-Negation)	0.2160	0.143	1.506	0.5587

Simple contrasts for path.

Instruction: No-instruction

Contrast	Estimate	SE	*z*.ratio	*p*.value
Noncontrastive - Contrastive	0.1734	0.222	0.782	0.4340

Instruction: Assertive-Assertive

Contrast	Estimate	SE	*z*.ratio	*p*.value
Noncontrastive - Contrastive	0.0346	0.227	0.153	0.8788

Instruction: Assertive-Negative

Contrast	Estimate	SE	*z*.ratio	*p*.value
Noncontrastive - Contrastive	-0.3781	0.224	-1.692	0.0907

Instruction: Negative-Assertive

Contrast	Estimate	SE	*z*.ratio	*p*.value
Noncontrastive - Contrastive	-0.0729	0.223	-0.327	0.7437

Instruction: Negative-Negative

Contrast	Estimate	SE	*z*.ratio	*p*.value
Noncontrastive - Contrastive	0.1121	0.221	0.506	0.6129

Table 2: Estimates for post hoc pairwise comparison for recall (Experiment 2)

Best model: *recall_score ~ instruction*path + (1|Subj) + (1|path)*

Supplementary materials

Simple contrasts for instruction.

Path: Noncontrastive

Contrast	Estimate	SE	*z*.ratio	*p*.value
(No-instruction) - (Assertion-Assertion)	-0.2837	0.131	-2.161	0.1947
(No-instruction) - (Assertion-Negation)	0.1683	0.127	1.329	0.6728
(No-instruction) - (Negation-Assertion)	-0.1084	0.129	-0.840	0.9182
(No-instruction) - (Negation-Negation)	0.0326	0.128	0.256	0.9991
(Assertion-Assertion) - (Assertion-Negation)	0.4520	0.130	3.478	0.0046
(Assertion-Assertion) - (Negation-Assertion)	0.1753	0.132	1.324	0.6761
(Assertion-Assertion) - (Negation-Negation)	0.3164	0.131	2.415	0.1113
(Assertion-Negation) - (Negation-Assertion)	-0.2767	0.128	-2.166	0.1925
(Assertion-Negation) - (Negation-Negation)	-0.1356	0.126	-1.074	0.8200
(Negation-Assertion) - (Negation-Negation)	0.1411	0.129	1.095	0.8090

Path: Contrastive

Contrast	Estimate	SE	*z*.ratio	*p*.value
(No-instruction) - (Assertion-Assertion)	-0.1703	0.160	-1.067	0.8237
(No-instruction) - (Assertion-Negation)	-0.2544	0.135	-1.886	0.3251
(No-instruction) - (Negation-Assertion)	0.1496	0.154	0.972	0.8680
(No-instruction) - (Negation-Negation)	0.1268	0.130	0.976	0.8662
(Assertion-Assertion) - (Assertion-Negation)	-0.0841	0.163	-0.517	0.9857
(Assertion-Assertion) - (Negation-Assertion)	0.3199	0.175	1.829	0.3565
(Assertion-Assertion) - (Negation-Negation)	0.2972	0.159	1.874	0.3313
(Assertion-Negation) - (Negation-Assertion)	0.4040	0.157	2.573	0.0754
(Assertion-Negation) - (Negation-Negation)	0.3812	0.134	2.854	0.0350
(Negation-Assertion) - (Negation-Negation)	-0.0228	0.153	-0.149	0.9999

Simple contrasts for path.

Instruction: No-instruction

Contrast	Estimate	SE	*z*.ratio	*p*.value
Noncontrastive - Contrastive	-0.1638	0.138	-1.185	0.2362

Instruction: Assertion-Assertion

Contrast	Estimate	SE	*z*.ratio	*p*.value
Noncontrastive - Contrastive	-0.0504	0.168	-0.299	0.7648

Instruction: Assertion-Negation

Contrast	Estimate	SE	*z*.ratio	*p*.value
Noncontrastive - Contrastive	-0.5865	0.140	-4.176	<0.0001

Instruction: Negation-Assertion

Contrast	Estimate	SE	*z*.ratio	*p*.value
Noncontrastive - Contrastive	0.0943	0.161	0.585	0.5586

Instruction: Negation-Negation

Contrast	Estimate	SE	*z*.ratio	*p*.value
Noncontrastive - Contrastive	-0.0696	0.137	-0.509	0.6106

Table 3: Experiment 1 - Classification of errors made during recall when an incorrect subaction was identified. The errors are categorized into three types:
a) Substitution: The correct subaction is replaced with a subaction associated with a different action, b) Other: An entirely unrelated subaction is performed, c) Omission: The subaction is skipped entirely.

Type	Number	Percentage (%)
**Substitution**	34	24.82%
**Other**	32	23.36%
**Omission**	71	51.82%
**Total error**	137	
**Total data points**	640	Accuracy=78.59%

Table 5: Random effects estimates and plots showing recall by subject and by item for Experiment 1 for path types: C1 (Action A), C2 (Action B), C3 (Action C), and C4 (Action D). Plots are shown for variables showing significant improvement in the model.

Groups	Term	Variance	Std. Dev.	Number of Levels
SubjID	(Intercept)	0.18060	0.4250	32
path	(Intercept)	0.02868	0.1693	4

Best model: *recall_score ~ instruction*path + (1|Subj) + (1|path)*

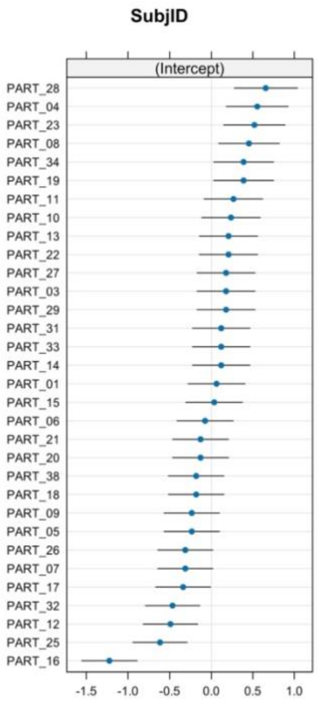

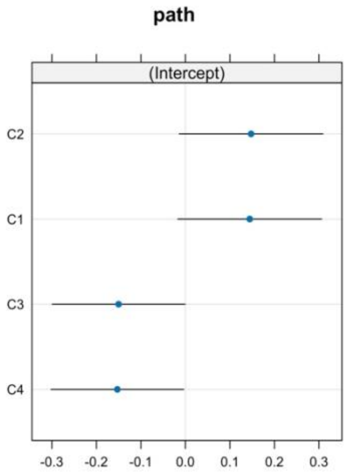

Table 4: Experiment 2 - Classification of errors made during recall when an incorrect subaction was identified. The errors are categorized into three types:

Type	Number	Percentage (%)
**Substitution**	26	20.00%
**Other**	35	26.92%
**Omission**	69	53.08%
**Total error**	130	
**Total data points**	720	Accuracy=81.94

Table 6: Random effects estimates and plots showing recall by subject and by item for Experiment 2. Plot is shown for variables showing significant improvement in the model.

Groups	Term	Variance	Std. Dev.	Number of Levels
SubjID	(Intercept)	0.148000	0.38471	40
path	(Intercept)	0.002252	0.04746	4

Best model:

*recall_score ~ instruction*path + (1|Subj)*

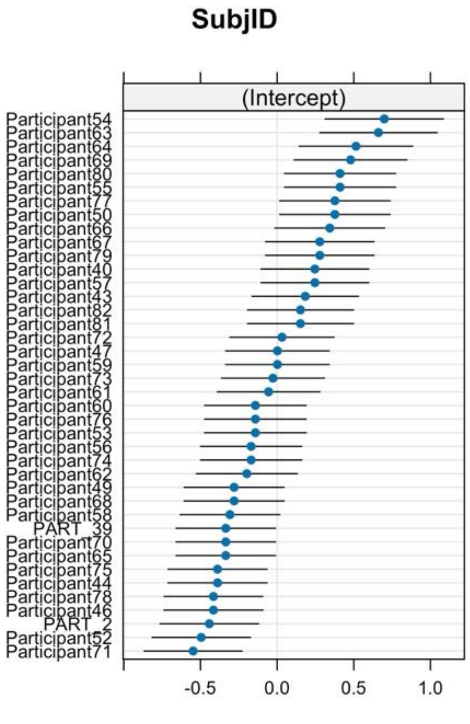

Table 7: Random effects estimates and plots for goal-looks by subject and by item for action types in figure 2: C1 (Action A), C2 (Action B), C3 (Action C), and C4 (Action D). Plots are shown for variables showing significant improvement in the model.

Supplementary materials

Groups	Term	Variance	Std. Dev.	Number of Levels
SubjID	(Intercept)	1.98660	1.4095	72
path	(Intercept)	0.09694	0.3113	4

Best model: *fixation_prop ~ mean_centered_recall + (1|Subj) + (1|action_type)*

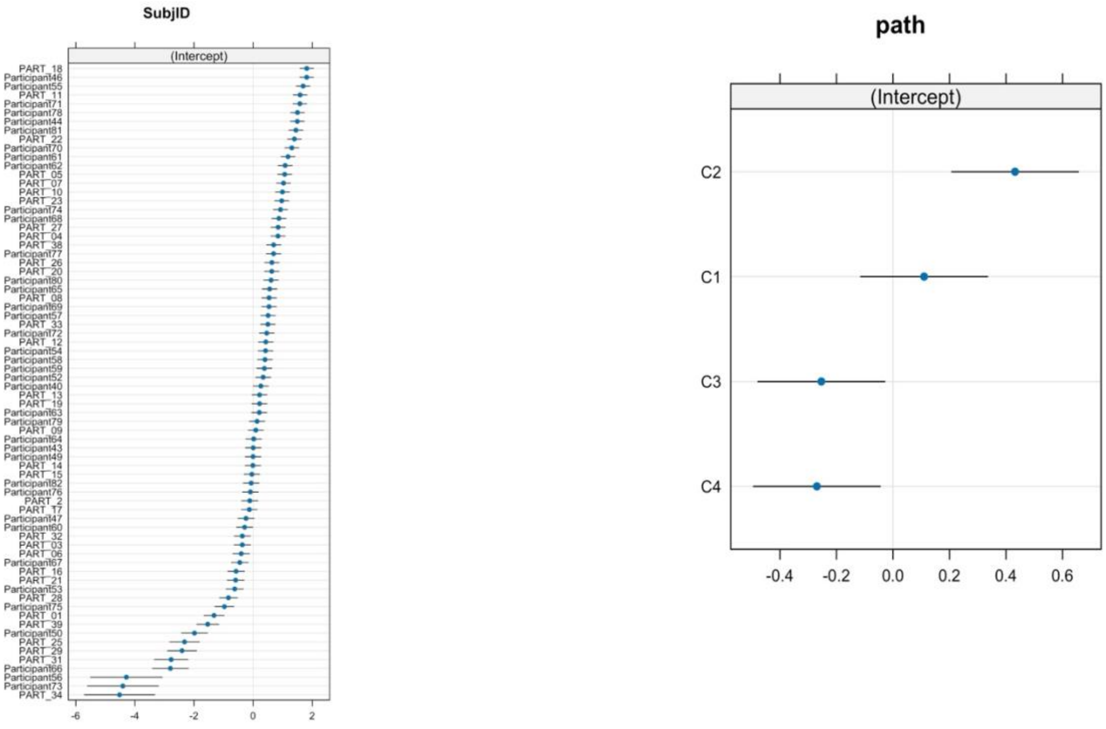

Figure 3: GAM difference smooth comparison between no-instruction and other instruction conditions as reported for Figure 3 and Figure 5 in the paper.

Supplementary materials

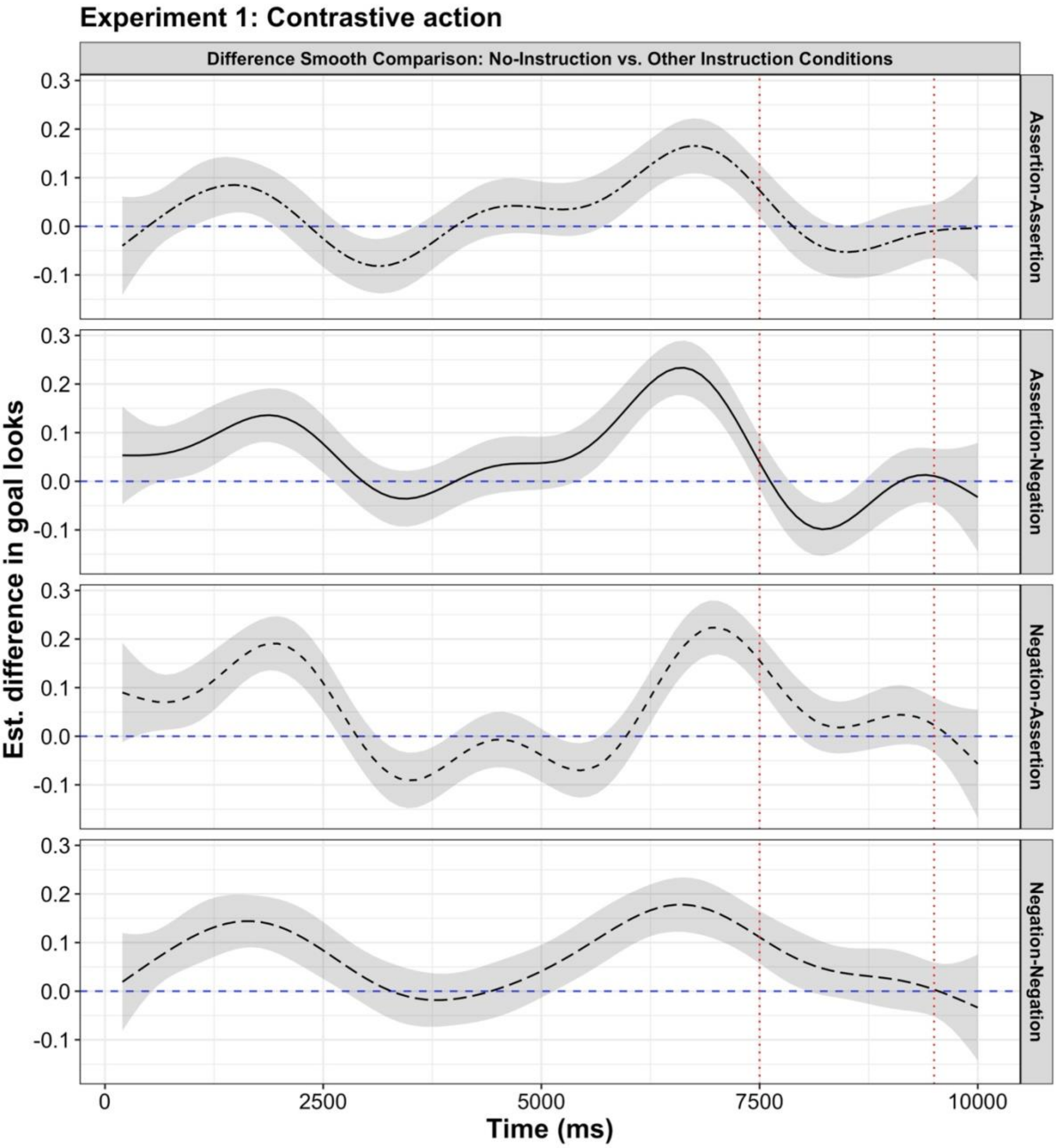

Supplementary materials

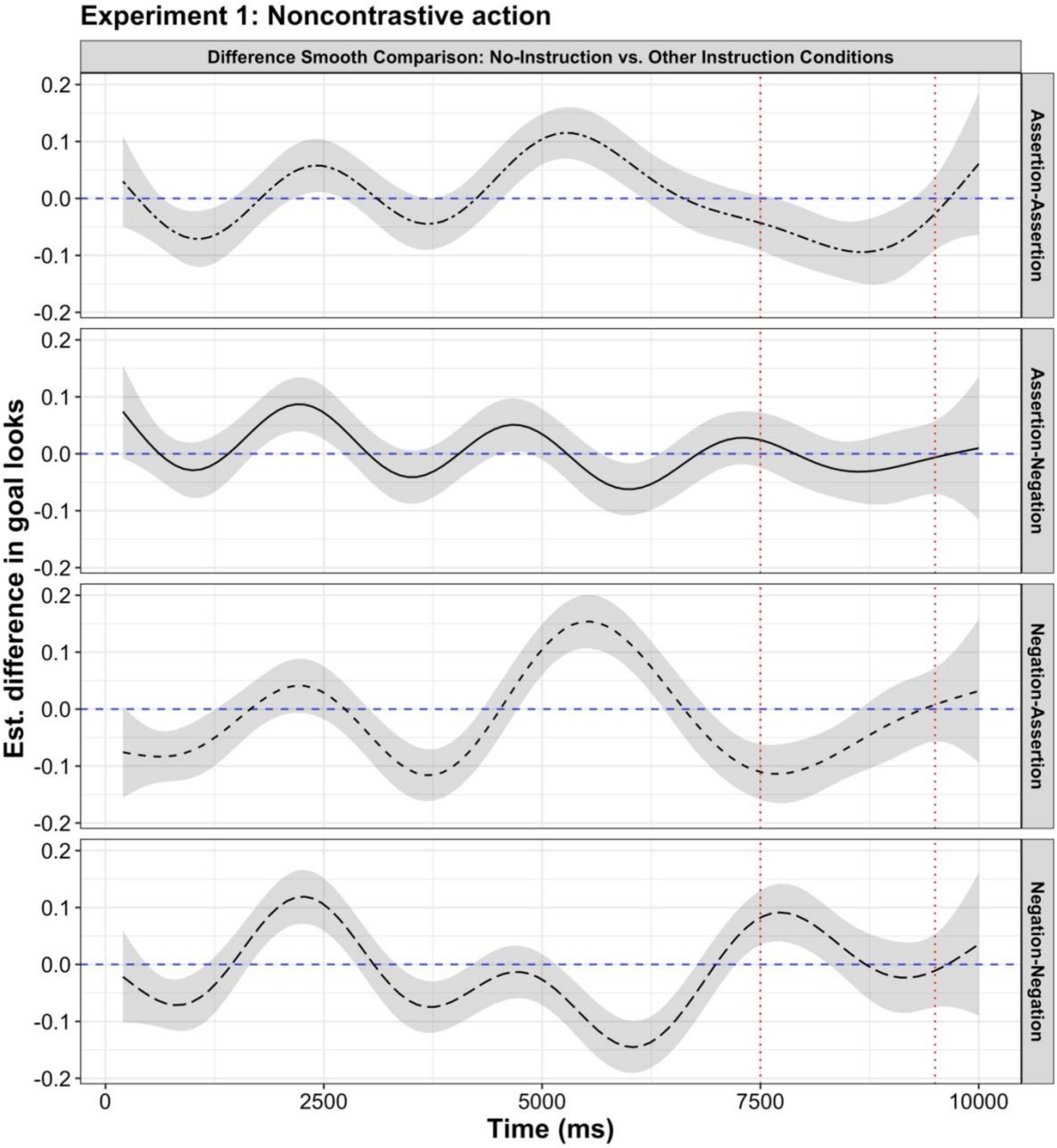

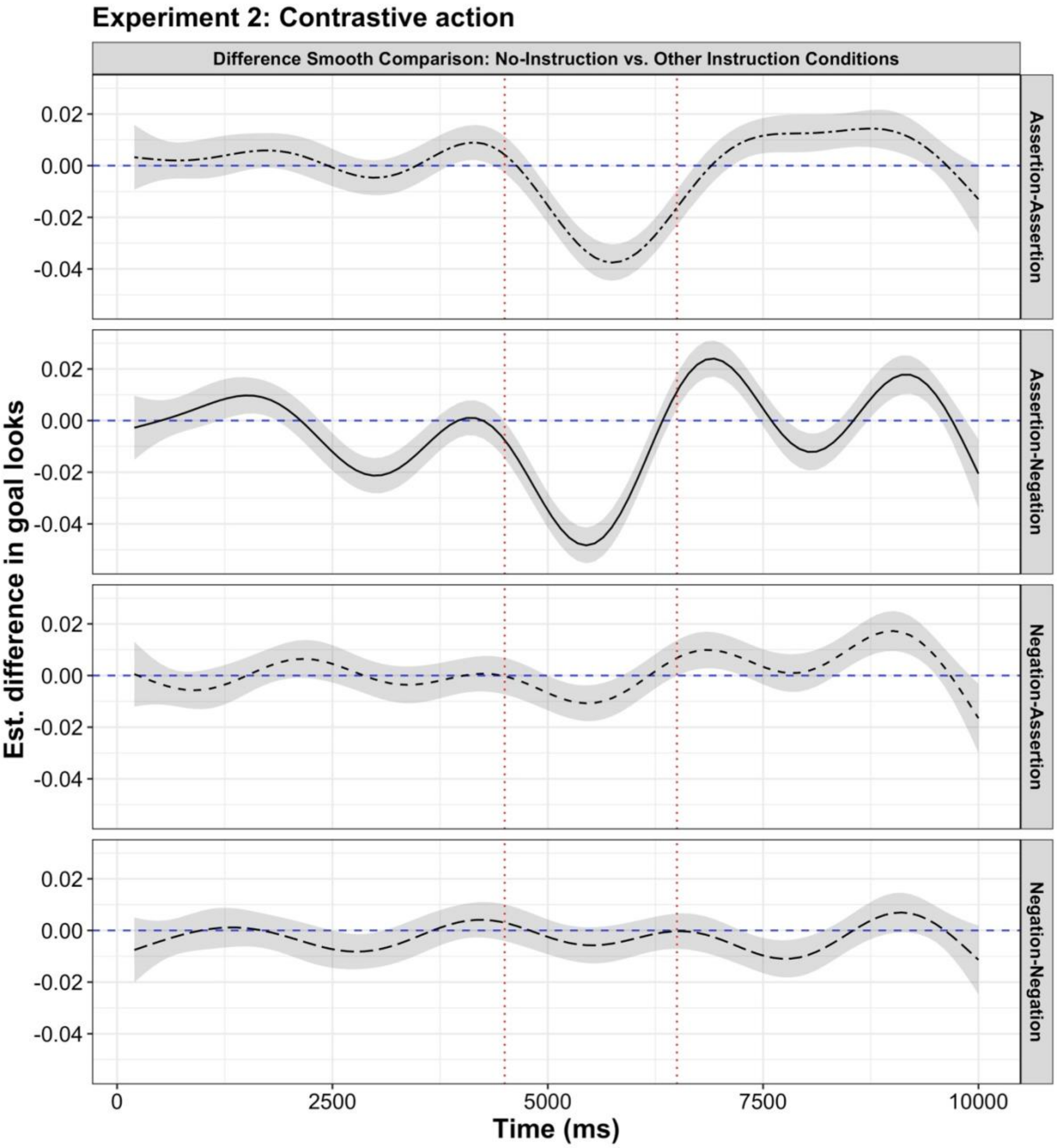
Experiment 2: Contrastive action
Difference Smooth Comparison: No-Instruction vs. Other Instruction Conditions
Assertion-Assertion
Assertion-Negation
Negation-Assertion
Negation-Negation
Est. difference in goal looks
0.02
0.00
-0.02
-0.04
0
2500
5000
7500
10000
Time (ms)

Supplementary materials

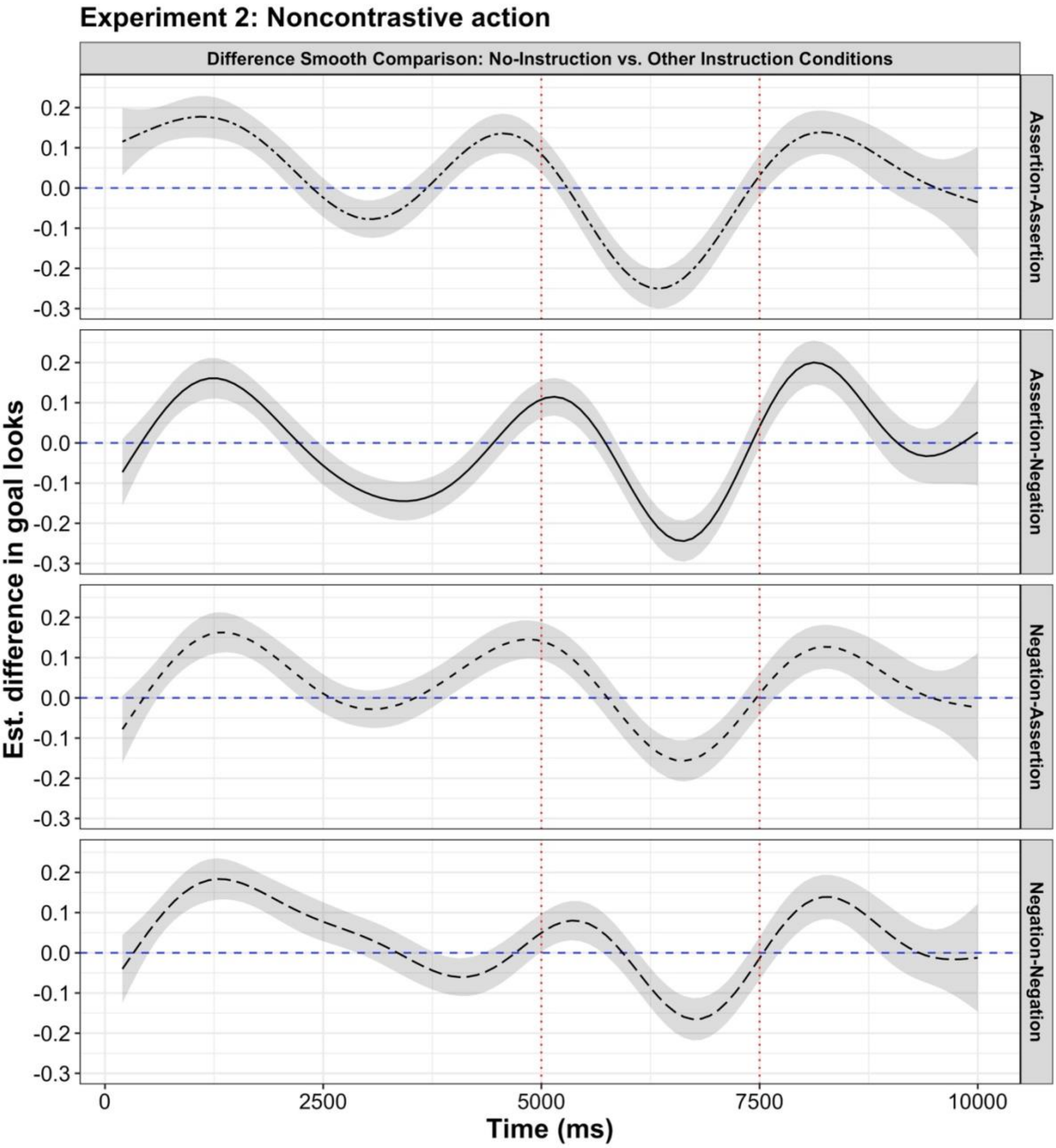

Supplement: Supplementary file 1 — Table S1: Estimates for post‐hoc pairwise comparison for recall (Experiment 1) Table S2: Estimates for post‐hoc pairwise comparison for recall (Experiment 2) Table S3: Experiment 1—Classification of errors made during recall when an incorrect subaction was identified Table S5: Random effects estimates and plots showing recall by subject and by item for Experiment 1 Table S4: Experiment 2—Classification of errors made during recall when an incorrect subaction was identified Table S6: Random effects estimates and plots showing recall by subject and by item for Experiment 2 Table S7: Random effects estimates and plots for goal‐looks by subject and by item for action types in Fig. 2: C1 (Action A), C2 (Action B), C3 (Action C), and C4 (Action D). Figure S3: GAM difference smooth comparison between no‐instruction and other instruction conditions as reported for Figs. 3 and 5 in the paper [file COGS-49-e70096-s001.pdf]
